# Mapping the evolution of cross-Strait relations via global news big data (2014–2023): An analysis integrating GDELT and machine learning

**DOI:** 10.1371/journal.pone.0342755

**Published:** 2026-02-13

**Authors:** Shengjie Shi, Belen Chen, Ziyi Guang, Derong Kong

**Affiliations:** 1 School of Journalism and Communication, Xiamen University, Xiamen, China; 2 School of Marxism, Guizhou Normal University, Guiyang, China; NingboTech University, CHINA

## Abstract

This study investigates global news media representations of cross-Strait relations from 2014 to 2023 using the GDELT database, framed within the mediatization of politics. Combining large-scale event data and computational text analysis, it offers a multi-level analysis of representational patterns, structural inequalities, and framing dynamics. Six indicators track longitudinal trends including four continuous indices and two event type distributions, show attention spikes during major political events and a discursive shift toward negative, conflict-focused coverage, even as low-intensity communicative events dominate. Structurally, a source-domain analysis of 50 high-impact outlets employs a six-dimensional Deviation Index to evaluate differences in visibility and event production preferences. Results reveal a concentrated discourse, with Western and Taiwanese media occupying more central positions in agenda-setting processes, with Mainland Chinese outlets appearing comparatively less visible. Textually, topic modeling uncovers five key frames, reflecting a discursive thematic evolution from event-driven, low-politics coverage to high-politics narratives emphasizing conflicts, ideological divides, and great-power rivalry. Complementary sentiment analysis of news headlines using large language model-based tools indicates the persistent dominance of negative actor-sentiment framing in global coverage. Overall, the study underscores an increasingly securitized and asymmetrical pattern of global media representation of cross-Strait relations. Theoretically, the study extends the applicability of mediatization of politics framework to the analysis of cross-Strait communication. Methodologically, it illustrates integrating large-scale event data with machine-learning techniques to examine international news framing in a highly politicized geopolitical context.

## 1 Introduction

Over the past decade, cross-Strait relations have become one of East Asia’s most closely watched geopolitical dynamics. The report titled “Preventive Priorities Survey 2025” published by the Council on Foreign Relations categorizes the Taiwan Strait region as “Tier I (High Priority) ” conflict zone with moderate likelihood of contingency and high impact on U.S. interests [1]. Strategic divergences in how China and the United States perceive the Taiwan issue have deepened. For the United States, Taiwan issue is primarily viewed it through geopolitics, ideology, and great-power rivalry. By contrast, China frames it as a matter of sovereignty, territorial integrity, and national rejuvenation [2]. Under conditions of intensifying U.S.-China strategic competition, cross-Strait relations have been increasingly understood and represented through global news coverage, where competing narratives are produced, circulated, and contested. Global media attention on cross-Strait relations has surged under the shadow of Sino-U.S. strategic rivalry, with both sides using the press to frame the Taiwan issue and seek to shape international perceptions.

Building on this discursive contestation, this study draws on the broader theoretical framework of mediatized politics, which posits that the public articulation of political processes and meanings are increasingly negotiated within and filtered through by media logic rather than simply reflected through news reporting [3,4]. This theoretical perspective emphasizes that political realities are continuously constructed, negotiated, and redefined through media communication. A growing body of literature indicates that contemporary politics, especially foreign policy and international relations, is deeply conditioned in its public presentation by media logic. For example, the mediatization of foreign policy highlights media logic conditions public prominence, narrative structure, and strategic responses as communicated and justified in the media of state actors [5]. From this perspective, the contestation over cross-Strait narratives is itself a process of mediatization—where journalistic routines and news values condition the framing of political understanding, policy discourse, and public sentiment across borders.

Empirical studies have begun to explore these dynamics. For instance, surveys conducted in major mainland Chinese cities reveal how public attitudes, psychological identification, and policy expectations toward Taiwan intersect with identity construction and national integration [6]. At the international level, Guo and Vargo analyzed a large amount of news data from international media including cross-Strait related reports to explore how news agendas flow across countries and regions [7]. Their findings suggest that cross-Strait relations function as a strategically salient node within the global information environment, being associated with international image construction and agenda-setting processes. Thus, the Taiwan issue transcends regional geopolitics, existing as a mediated political phenomenon whose global perception embedded within complex transnational communication structures.

Historical episodes further illustrate how media, public opinion, and political identity converge to contribute to the formation of cross-Strait discourse. In 2014, Taiwan’s Sunflower Movement illuminated deep social debates over economic policy, political identity, and the island’s future status. C. K. S. Wu analyzed how public opinion influenced the policy decisions of elites and analyzed the public’s profound participation in economic policies such as Cross-Strait Service Trade Agreement (CSSTA) and political future status in this social movement [8]. Researchers have also pointed out that the “China factor” became a key discourse. It not only involved opposition to economic policies, but was also directly related to the construction of political identity and Taiwan’s future status discourse [9]. These dynamics were further embedded in an international context as external actors, the United States, became increasingly visible in Taiwan-related discourse. Following the 2016 power transition in Taipei and the transition in the U.S. administration, new divergences emerged as both sides across the Strait advanced contrasting narratives on sovereignty and security. In this context, global media coverage has become a key arena where international public opinion, domestic political debates, and great-power rivalries converge.

This study reconceptualizes cross-Strait relations not as a static geopolitical standoff, but as a dynamic, mediated process of meaning-production. Rather than treating media reports as neutral reflections of political events, this approach understands them as constitutive practices of public meaning-making that frame how “relations” are perceived, problematized in the public sphere.

Despite these advances, existing research remains methodologically and analytically fragmented. Traditional content analyses are frequently confined to single‑country outlets or limited time spans. Effendi synthesized media coverage frameworks such as frame analysis, agenda setting, and discourse analysis [10]. While these studies offer valuable qualitative insights, they cannot adequately capture the longitudinal evolution, global diffusion, and structural inequality of media narratives. Similarly, research examining self-censorship and reporting tendencies in cross-Strait media from 2010 to 2016 relies primarily on process tracing and manual content analysis [11], which constrains scalability and cross-national comparability. Qualitative case studies are vulnerable to selection bias, and small-scale quantitative coding struggles to accommodate the volume, diversity, and multilingual nature of global news coverage.

To bridge these empirical and theoretical gaps, this study harnesses the Global Database of Events, Language, and Tone (GDELT) to construct a data-driven, longitudinal, and cross-national analytical framework. GDELT, which automatically collects and codes millions of news items in real time, allowing for the application of topic modeling and sentiment analysis to reveal macro-level patterns and frames. By integrating time-series indices (Attention, Balance, Influence, Tone), event coding schemes (QuadClass, EventRootCode), and deviation metrics across leading outlets, this study systematically examines how cross-Strait relations are monitored, structured, and framed worldwide.

This study extends previous work in two critical ways. First, it transcends the methodological limitations of manual content analysis by leveraging the GDELT big-data infrastructure, enabling a systematic, longitudinal, and multi-dimensional observation of global narrative patterns. Second, it advances the mediatized politics framework by empirically mapping the structural transformation of cross-Strait relations from an issue of “low politics” (economic exchange) to one of “high politics” (strategic rivalry) across the global information ecosystem. By doing so, this research provides a comprehensive, data-driven map of how international political meanings are constructed and contested within the global news system.

Accordingly, this study addresses three research questions: (1) How are cross-Strait relations monitored and evaluated in global media through large-scale event frequency, interaction intensity, and tonal indicators? (2) Which countries’ or regions’ media outlets play a dominant role in shaping the global narrative, and how do their representations diverge across multiple dimensions? (3) What thematic trajectories characterize the most globally visible coverage of cross-Strait relations, and how do emotional and evaluative frames evolve over time?

By answering these questions, the study offers three major contributions. First, it advances political communication research by demonstrating how big-data infrastructure, combineing GDELT event coding, topic modeling (LDA), and LLM-based sentiment analysis, can be leveraged to conduct systematic, longitudinal, and cross-national analyses of international media narratives. Second, it empirically elucidates how global media representations of cross-Strait relations have shifted from an emphasis on “low politics” to a focus on “high politics”. Third, it extends the theoretical implications of mediatized politics by empirically illustrating how global media not only transmit but actively constitute the meanings of cross-Strait relations. In doing so, this study moves beyond single-country perspectives and static case analyses, offering a more comprehensive understanding of how international political realities are constructed through global media systems.

## 2 Literature review

Adopting the meta-theoretical framework of mediatized politics, this study conceptualizes cross-Strait relations as continuously shaped and reproduced through media practices. From this perspective, how media representation, visibility, framing, and sentiment operate within global communication systems is essential to grasping the mediated construction of geopolitical reality. Accordingly, mediatized politics serves as the analytical foundation for examining the construction, negotiation, and redefinition of cross-Strait dynamics across three interconnected dimensions: symbolic representation, structural inequality, and computational framing. The following sections synthesize existing scholarship concerning: the representation of cross-Strait relations in global media, imbalance in the global communication system and internal differentiation among high-impact media, and the evolution of framing research from interpretive to computational approaches.

### 2.1 The representations of cross-Strait relations by global media

Within the framework of mediatized politics, the global media are active institutions that influence the symbolic construction how political relations are symbolically constructed and publicly perceived. This section reviews how news media reconstruct cross-Strait relations, revealing the mediated processes through which political meanings, identities, and interactions are produced.

Over the past two decades, the role of news media in shaping cross-Strait relations has occupied a central position in political communication research. Scholars concur that the public perceives cross-Strait relations through their continuous reconstruction within news production and circulation. Through selective coverage, narrative framing, and evaluative orientation, the media embed cross-Strait relations within specific structures of meaning, conditioning public perceptions of the nature, risks, and prospects of interactions.

Extant empirical literature has predominantly focused on event-driven analyses. These studies scrutinize key political, economic, or social milestones, analyzing how media construct relational portrayals through issue framing. Pan and Qiao identified marked differences in news selection and narrative strategy, in their comparison of Lien Chan’s “Journey of Peace” coverage, revealing distinct political positioning across media contexts [12]. Similar studies have analyzed of Mainland entrepreneurs’ investments in Taiwan [13] and the “Xi-Ma Meeting” [14], which demonstrate the systematic features of issue selection and frame construction in news coverage.

Subsequent scholarship shifted from specific events to overall relational images constructed by the media. These works suggest that media do not report individual events in isolation but, through sustained and iterative reporting practices, reinforce a stable cognitive structures of cross-Strait relations within journalistic narratives. Ruiz Casado and Lock demonstrated that visual media reproduce normalized perceptions of cross-Strait relations, even when discussing other geopolitical actors [15].

Meanwhile, other studies have explored the linguistic and discursive dimensions of representational practices. The analysis of Taiwan-related reports in English-language media demonstrates that subtle variations in lexical choice, syntactic structure, and metaphor usage correspond to proximity or distance from either Beijing’s or Taipei’s stance, especially during political crises and elections [16].

Beyond textual and discourse analysis, current scholarship connects media representations with public perception and political behavior, thereby integrating socio-psychological and behavioral dimensions. Empirical studies suggest that mediated narratives on cross-Strait relations can shape public attitudes and patterns of party support through electoral framing, risk narratives, or economic expectations. For instance, Hsu linked shifts in public opinion during Taiwan’s “nine-in-one” local elections to the issue structure of Mainland-related coverage, noting that electoral representations are associated with distinct mobilizing effects [17]. Similarly, surveys of Mainland Chinese citizens indicate that attitudes toward unification are shaped by the state’s discursive system, policy narratives, and information ecology [18]. Studies in Taiwan and Hong Kong likewise reveal that political identity and recent events significantly influence perceptions of Mainland China, underscoring the profound effects of media coverage and the information environment on public perception [19].

Crucially, the symbolic construction of cross-Strait relations is a transnational phenomenon. As a geopolitically sensitive issue, cross-Strait relations attract consistent attention from third-party media. Through a comparative analysis of China-U.S. media coverage, Lu found that ideological orientations shape the narrative structures surrounding cross-Strait relations [20]. However, some studies point out that certain third-party media do not adopt an adversarial stance. For instance, Chen and Nylén suggest that while Japanese mainstream outlets cannot fully detach from ideological influences, they generally tend to emphasize stability and restraint [21,22]. Similarly, Central American media often frame the cross-Strait issue within an “economic incentives” narrative, revealing a distinctive global perception pattern [23].

Despite this thematic and methodological expansion, existing research still retains significant structural limitations. First, the predominance of event-driven analysis limits insights into the long-term evolution of cross-Strait representations. Second, scope is usually restricted to limited geographic or institutional clusters, lacking a systematic examination within the global news system. Third, traditional content and discourse analysis methods struggle to accommodate multilingual, large-scale news corpora, hindering a comprehensive depiction of global dynamics.

Against this backdrop, emergent research leverages large-scale news data to systematically measure the media representation of international relations. Databases such as the Global Database of Events, Language, and Tone (GDELT) enable coding of actors, interactions, and tone, facilitating analysis of how states and regions are portrayed globally. Prior work shows that indicators based on event intensity, interactional directionality, and tone can trace the trajectory of bilateral relations over extended periods [24,25]. However, most GDELT-based studies focus on general international conflict, with limited attention to the historical and political context of cross-Strait relations.

While prior studies have revealed the media’s multifaceted role, they lack a systematic framework for analyzing these representations on global and longitudinal scales.This study addresses this gap by leveraging global news data to systematically measure the representational intensity, interactional structure, and emotional orientation of cross-Strait relations, thereby providing novel empirical evidence of their mediatized evolution.

### 2.2 Global visibility imbalance and internal differentiation of high-impact media

Under mediatized politics, global communication imbalance is a reflection of how political power intertwined with media logic to influence the perceived visibility and discursive legitimacy of international actors. Such inequalities reveal the constitutive role of media institutions in structuring, rather than merely transmitting, geopolitical perceptions.

Scholars have long documented a persistent asymmetry in global information flows, where visibility and agenda-setting power are monopolized by a few dominant media organizations situated in developed economies. Schiller argued that Western control over information flows marginalizes non-industrialized nations seeking economic and social sovereignty [26]. Shamsuddin also emphasized the persistent marginalization of certain nations within the global communication order [27]. This reinforces a structural inequality where a few Western media exercise disproportionate control over international visibility of world events. Such unidirectional flows deprive non-Western actors of discursive agency, solidifying a hierarchical international information order.

This hierarchy persists even in demostic digital spaces. Golan found that international news flows on social media largely mirror institutional core-periphery structures, despite the presence of non-institutional actors [28]. Gunaratne argued that economic power blocs, rather than nation-states, drive this informational capitalism [29]. Consequently, visibility serves as symbolic capital that reproduces power hierarchies across media systems.

However, assessing imbalance through visibility metrics is insufficient. Media organizations with comparable influence may adopt distinct interpretive frameworks. Such internal heterogeneity among “elite media” indicates that imbalance manifests both as external exclusion and internal narrative divergence among central actors.

Political and ideological orientations shape coverage, producing systematic variation in geopolitical framing. Even media outlets with comparable global reach display substantial divergence in their reporting practices [30]. In the cross-Strait context, Western media often emphasize strategic competition and security crisess [31], whereas mainland Chinese outlets prioritize national sovereignty and territorial integrity [32]. Conversely, Taiwanese media reflect internal ideological fragmentation and audience segmentation.

Moreover, global media operate within a transnational network of mutual influence. Structural news flow studies suggest that visibility concentration does not guarantee narrative homogeneity. The structural characteristics of cross-media influence among major news organizations can affect coverage diversity. While high network density accelerates narrative convergence, network clustering allows outlets to maintain distinct interpretive frames [33]. Therefore, understanding global news production requires examining the internal relational structures of elite media, beyond mere attention metrics.

News framing thus embodies political orientation and power dynamics within global communication structures. Horne et al. demonstrated that while content homogenizes within tightly connected media communities, it diverges significantly across them, even when covering identical events [34]. This demonstrates that narrative convergence coexists with structural differentiation. Similarly, large-scale corpus analyses reveal that internal thematic preferences drive persistent framing competition among dominant outlets [35].

Therefore, to gain deeper insight into how the most influential media clusters construct narratives of cross-Strait relations, this study examines not only visibility concentration and geographic distribution of reporting but also internal structures and narrative divergences among these core media actors. This dual focus on external concentration and internal divergence provides a nuanced lens for examining informational power in geopolitical discourse.

Particularly in sensitive contexts involving national identity, policy competition, and struggles over discursive power, the framing and affective orientations adopted by different media toward the same event often reflect deeper layers of political identity and ideological divergence [36]. By quantifying both regional imbalances and internal heterogeneity, this study explores the structural coexistence of influence and divergence within the contemporary international communication system.

### 2.3 News framing in mediatized politics: from interpretive to computational

Framing research, rooted in Goffman’s concept of organizing social reality [37], is transitioning from interpretive categories toward computable structures under mediatized politics.

Since Entman’s four functions of framing [38], scholars have recognized framing as an active process of constructing political meaning through symbolic selection. In mediatized contexts, framing serves as a generative mechanism that structures relational meanings in political communication. As media outlets transition from observers to constitutive actors, research has shifted from classifying static frames to examining how framing conditions the patterns of visibility and interpretation. Consequently, framing acts as the mediator through which the representation of political relations is mediatized, evaluated, and narratively institutionalized.

Computational advances have shifted framing analysis toward operational structural pathways. First, topic models have been widely used as approximate tools for issue-frame structures. Related studies argue that the repeated co-occurrence and semantic aggregation of issues in news texts often constitute stable narrative patterns, which, although not directly equivalent to frames, can reveal structural traces of framed meanings. The structural topic model (STM) provides a crucial statistical foundation for this approach, enabling researchers to identify differences in issue combinations across contexts while controlling for metadata [39]. Subsequent studies demonstrate that the thematic structures of news issues are closely related to the selective presentation of frames, reflecting how media construct the meaning space of political events through persistent issue co-occurrences [40,41]. Second, sentiment analysis has evolved from a subsidiary metric to a core evaluative dimension of framing [42,43]. In geopolitical reporting, sentiment polarity often correlates with frames of threat or cooperation, influencing public attitudes and the communicative environment of interstate relations. By measuring sentiment in textual positions such as headlines and leads, researchers can capture systematic differences in the evaluative dimensions of news narratives, which is particularly critical for long-term dynamic analyses of bilateral relations.

Recent scholarship integrates topic prevalence and sentiment polarity to identify composite frames, such as securitization or cooperation, without manual coding [44,45]. This methodological innovation preserves the interpretive depth of framing while ensuring replicability in large-scale datasets.

Although computational communication has expanded the empirical dimensions of framing research, limitations in longitudinal and relational measurement persist. First, most studies focused on single issues or short-term events, like elections or crises, lacking systematic depictions of long-term dynamics of evolution and relational structures [46]. In transnational contexts, interstate relations continuously produced through long-term media representations and narrative repetitions. Second, research using event data or sentiment indices (e.g., GDELT or ICEWS), often treats news as external observations, neglecting framing’s structural role in political meaning production [47]. Furthermore, framing is rarely positioned as an analytical intermediary, often being reduced to an ancillary variable. Under mediatized politics, the meaning of political events are encoded, disseminated, and socially perceived through the intermediary of news framing. Addressing these gaps, this study employs framing as an analytical intermediary, integrating topic modeling and sentiment analysis to systematically measure the long-term structural evolution of cross-Strait relations. This approach responds to the theoretical significance of framing while revealing the dynamic features of global geopolitical representation.

## 3 Materials and methods

### 3.1 Data source

The GDELT (https://www.gdeltproject.org/) encompasses news reports from over 100 languages in global mainstream and online media since 1979. The database extracts core events from news reports and codes each event using 58 fields.

This article implemented custom Python scripts to retrieve all GDELT 1.0 event tables from January 1, 2014 through December 31, 2023, extending the query window to February 2024 to accommodate potential reporting lags but ultimately restricting the analytic sample to events dated between January 2014 and December 2023. Events were filtered for cross-Strait relevance by requiring that either Actor1CountryCode or Actor2CountryCode be “CHN” (Mainland China) or “TWN” (Taiwan Region). This process yielded 395,465 cross-Strait events in total, of which 213,041 list Mainland China as Actor 1 and 182,424 list Taiwan Region as Actor 1.

### 3.2 Research methods

#### 3.2.1 The representation of global media in reporting cross-Strait relations.

To examine how global news media represented the evolution of cross-Strait relations between 2014 and 2023, this study operationalizes media representations as aggregated patterns of attention, interactional balance, evaluative impact, and tone embedded in large-scale news reporting, rather than as direct reflections of underlying political reality. This operationalization is consistent with prior research that treats international relations as being mediated and reconstructed through news production processes rather than merely reported.

Drawing on structured event information from the GDELT, six dimensions are employed to capture different aspects of global media representations of cross-Strait relations.

*(a) Attention Index.* Let *A(t)* denote the attention index, *NCT(t)* is the number of events in month *t* for events initiated by Mainland China toward Taiwan Region and *NTC(t)* is the number of events in month *t* for events initiated by Mainland China toward Taiwan Region.


$$A(t)=𝐥𝐧(1+N_{CT}(t)+N_{TC}\left(t\right))$$ (1)

The Attention Index captures the overall salience of cross-Strait relations in global news coverage, reflecting the degree to which cross-Strait interactions enter the global media agenda in a given period. Logarithmic transformation is applied to reduce the influence of extreme values during periods of heightened media activity.

*(b) Balance Index.* Let *B(t)* denote the Balance Index, *NCT(t)* and *NTC(t)* is the same meaning in the formula (1). If *B(t)*=0.5, it means there is a perfectly balanced coverage between the two sides cross the Strait.


$$B(t)=\frac{N_{CT}\left(t\right)}{N_{CT}(t)+N_{TC}\left(t\right)}$$ (2)

Deviations from this value indicate asymmetric attribution of initiative or agency in media representations, highlighting which side is more frequently portrayed as the actor in cross-Strait events.

Unlike the impact and tone indices, which rely on continuous event attributes and weighted aggregation, the Balance Index captures the structural directionality of media coverage by measuring the proportion of Mainland China-to-Taiwan Region events relative to total cross-Strait interactions. As this indicator is intended to describe the observed configuration of media representations rather than to estimate an underlying latent distribution, the main analysis reports the original monthly proportions. Bootstrap resampling is therefore not required for substantive interpretation and is used only as a robustness check in supplementary analyses.

*(c) Impact Index.* Let *I(t)* denote the Impact Index of cross-Strait relations, *NumMentionsi* the *NumMentions* (weight) for event *i*, and *GoldsteinScalei* the *GoldsteinScale* for event *i*. In *t*, the weighted average is given by:


$$\overset{―}{I}(t)=\frac{\sum_{i\in t}{{GoldsteinScale}_{i}\times NumberMentions}_{i}}{\sum_{i\in t}{NumberMentions}_{i}}$$ (3)

Because *GoldsteinScalei*∈[-10,10], affine mapping *I(t)* translates the original [−10, 10] range to [0,1] enabling direct comparability with other normalized metrics.


$$I(t)=\frac{\overset{―}{I}(t)+10}{20}$$ (4)

To address potential noise arising from automated event coding, media duplication, and weighting effects in GDELT data, uncertainty estimation is conducted using nonparametric bootstrap resampling. Specifically, events within each month are resampled with replacement 2,000 times. For each resampled event set, the Impact Index are recalculated based on weighted aggregation of event attributes. The resulting bootstrap distributions are then used to derive monthly point estimates (means) and 95% confidence intervals. This procedure allows temporal peaks and troughs to be interpreted in terms of their statistical stability rather than as isolated point estimates.

*(d) Tone Index.* Let *T(t)* denote the tone of global reporting of cross-Strait relations, *NumMentionsi* the *NumMentions* (weight) for event *i*, and *AvgTonei* the average tone for event *i*. In *t*, the weighted average is given by:


$$\overset{―}{T}\left(t\right)=\frac{\sum_{i\in t}{{AvgTone}_{i}\times NumberMentions}_{i}}{\sum_{i\in t}{NumberMentions}_{i}}$$ (5)

Because *AvgTonei*∈[-10,10], affine mapping *I(t)* translates the original [−10, 10] range to [0,1] enabling direct comparability with other normalized metrics.


$$T(t)=\frac{\overset{―}{T}(t)+10}{20}$$ (6)

The same event-level bootstrap resampling strategy is applied to the Tone Index to ensure methodological consistency across indices. Using identical monthly resamples as Impact Index, the Tone Index is recalculated for each bootstrap iteration based on weighted average tone scores, and 95% confidence intervals are constructed from the resulting distributions.

*(e) The Evolution of QuadClass and EventRootCode.* GDELT assigns each event to one of four QuadClasses: Verbal Cooperation, Material Cooperation, Verbal Conflict, Material Conflict. For each month *t*, count frequencies *Q(t)=[Q1, Q2, Q3, Q4]*. CAMEO’s 20 category EventRootCode taxonomy covers a wide spectrum of political and social behaviors. Monthly frequency vector *E(t)∈N20*.

These categorical distributions are used to examine structural changes in the types of interactions emphasized by global media, rather than to estimate continuous trends; therefore, uncertainty estimation is not applied at this stage.

#### 3.2.2 Identifying high-impact media outlets and conceptualizing the deviation index.

To determine which news organizations wield high influence in cross-Strait reporting, this study leverages GDELT’s SourceURL field. Each news event in GDELT is tagged with a URL. Parsing these URLs yields the hostname, which in turn serves as a proxy for the media outlet and its country/region of origin. This organizational-level identification is consistent with prior large-scale studies of international news flows and enables systematic comparison among media actors embedded in the global news ecosystem.

A cutoff of 1,000 total cross-Strait event mentions over the 2014–2023 period was chosen to distinguish outlets with sustained engagement from those with sporadic coverage. The initial domain list comprised 51 that exceeded the threshold. Two URLs (yahoo.com and news.yahoo.com) represent the same corporate entity and their counts were merged, yielding the final set of 50 high-impact outlets.

The analytical focus of this study extends beyond the concentration of global visibility to the internal differentiation among these already highly visible outlets. As discussed in Section 2.2, when global news attention is disproportionately concentrated among a limited set of media organizations, the extent to which these outlets converge or diverge in their reporting structures becomes theoretically consequential for understanding the production and fragmentation of global discourse. The Deviation Index is designed to capture this form of structural differentiation in reporting profiles, rather than to measure influence, ideological bias, or normative stance.

Conceptually, the Deviation Index operationalizes deviation as a multidimensional distance between an outlet’s longitudinal coverage pattern and the aggregate distribution produced by the global news system. The global baseline thus serves as a relational reference point, representing an emergent equilibrium of international reporting rather than a normative standard. Deviation is therefore analytically neutral: a higher index value indicates consistent divergence from the global baseline, not greater bias or stronger influence.

The measurement framework applied to fifty high‑impact outlets comprises six complementary dimensions: four continuous indices (EventCount, BalanceIndex, ImpactIndex, ToneIndex) and two categorical distributions (QuadClass and EventRootCode). Each dimension captures a different operational layer at which editorial differentiation may manifest, including reporting intensity, directional balance, event salience, emotional orientation, and the distribution of interaction types.

For continuous dimensions, each outlet’s monthly time series is compared to the global media baseline using the root-mean-square error (RMSE). To ensure scale invariance and comparability across dimensions, RMSE values are normalized by the baseline’s sample standard deviation σ, yielding σ-normalized RMSE (σ-NRMSE). These values are then standardized across outlets (Z-scores) and transformed using a logistic sigmoid function to produce bounded deviation scores in the interval (0, 1). This transformation ensures that no single continuous dimension dominates the composite index due to scale, variance, or outlier effects.

Categorical dimensions are modeled as monthly probability distributions over the four QuadClass categories and twenty EventRootCodes, respectively. Each outlet’s histogram is compared to the baseline distribution using the Jensen-Shannon distance, which symmetrizes and smooths the Kullback-Leibler divergence and yields values in the range [0, 1]. This approach captures divergence in the structural composition of reported interactions, rather than differences in volume alone.

The six dimension-specific deviation scores are aggregated using an unweighted arithmetic mean to form the overall Deviation Index. This aggregation is intended as a descriptive summary of overall editorial differentiation, rather than as an estimate of a latent variable. Equal weighting reflects a deliberate theoretical stance: no a priori assumption is made regarding the relative primacy of any single dimension in defining structural differentiation among high-impact media. Assigning equal weights thus avoids imposing normative or theoretically underdetermined hierarchies among reporting features and provides a transparent and replicable basis for cross-outlet comparison.

To address the concern that equal weighting constitutes a substantive analytical choice rather than a neutral default, this study explicitly evaluates alternative aggregation strategies and tests the sensitivity of the composite index to its weighting and metric assumptions. First, an empirically derived weighting scheme based on principal component analysis (PCA) was applied to the six deviation dimensions. The resulting weights are broadly balanced across dimensions, with no single metric exhibiting clear dominance, and the PCA-weighted composite index displays an almost perfect rank-order correspondence with the equal-weighted index (Spearman’s ρ = 0.991, p < 0.001). Second, a leave-one-dimension-out sensitivity analysis was conducted by iteratively recalculating the composite index after removing each dimension in turn. In all cases, the resulting indices remain highly correlated with the full specification (Spearman’s ρ ranging from 0.937 to 0.999), indicating that the overall structure of outlet differentiation is not driven by any single component. Third, robustness checks were performed by substituting alternative distance and error metrics in the construction of the deviation measures, including replacing RMSE with MAE for continuous dimensions and Jensen–Shannon distance with the Hellinger distance for categorical distributions. The composite index derived from these alternative specifications exhibits strong rank-order stability relative to the baseline index (Spearman’s ρ = 0.934), limited average rank displacement, and a high degree of quartile consistency across outlets. These analyses suggest that while equal weighting is a theoretically explicit baseline choice, the substantive conclusions drawn from the Deviation Index are robust to alternative weighting schemes, dimensional exclusions, and measurement specifications, and therefore reflect a stable underlying pattern of editorial differentiation among high-impact media outlets.

Accordingly, the Deviation Index should be interpreted as an integrative measure of how distinct a given high-impact outlet’s reporting profile is relative to the global news system as a whole. By focusing on relative deviation rather than absolute dominance, the index enables systematic comparison among media organizations originating from diverse institutional, political, and regional contexts, thereby empirically linking global visibility concentration to internal differentiation among media elites.

#### 3.2.3 Textual analysis of significant news events with machine learning.

To operationalize news framing under conditions of mediatized politics, this study conducts a micro-level textual analysis of significant cross-Strait news events by integrating topic modeling and sentiment analysis. Following the computational framing approach outlined in Section 2.3, framing is conceptualized as a combination of issue emphasis and evaluative orientation, which can be approximated through the joint analysis of topic structures and headline sentiment.

Machine learning techniques are applied to conduct a micro-level textual analysis, which comprises three key steps.

*(a) Selection of Textual Samples.* News events are ranked based on the NumMentions metric, and the top 100 cross-Strait news reports per year are selected as the sample of significant events, totaling 1,000 reports over the ten-year period. 50 reports are selected from the “Mainland China-Taiwan Region” (CHN-TWN) group and 50 from the “Taiwan Region-Mainland China” (TWN-CHN) group annually. The full texts of these reports are retrieved via the SourceURL field. To ensure sample integrity, duplicate reports are systematically removed and replaced to maintain a consistent sample size of 100 reports per year. This selection strategy prioritizes events that are most salient within the global media agenda, thereby aligning the analysis with the mediating role of news coverage emphasized in mediatized politics. The rationale for focusing on high-salience reports is grounded in mediatized politics, where global political perception is primarily structured by highly visible news events rather than the total volume of sporadic reporting.

*(b) Topic Model Analysis.* Prior to topic modeling, all news texts undergo a standardized preprocessing pipeline designed to enhance semantic coherence while preserving issue-relevant expressions in cross-Strait reporting.

First, texts are tokenized at the word level using a rule-based regular expression tokenizer. All tokens are converted to lowercase, and punctuation, numerical strings, and non-alphanumeric characters are implicitly removed through tokenization rules. Standard English stop words are excluded using an externally defined stop-word list, which additionally incorporates corpus-specific functional terms (e.g., “a/an,” “according,” “anything”) that do not contribute to topical differentiation.

Second, to preserve semantically meaningful expressions frequently used in cross-Strait discourse, a predefined list of domain-specific multiword expressions is incorporated prior to tokenization. These include commonly occurring phrases such as “Hong Kong,” “Taiwan Independence,” “One China policy,” and “South China Sea,” and treated as single lexical units during modeling. This step mitigates semantic fragmentation and improves topic interpretability.

Third, lemmatization is applied using WordNet-based lemmatization to reduce inflectional variation while preserving part-of-speech consistency. After preprocessing, separate Latent Dirichlet Allocation (LDA) models are estimated for each yearly corpus, using the Gensim LdaMulticore implementation with 500 passes (iterations) to ensure model convergence. To improve computational efficiency, parallelized inference is employed with eight worker processes, and a fixed random seed (random_state = 42) is specified to ensure reproducibility.

Rather than selecting a single optimal topic solution a priori, LDA models are estimated across a predefined range of topic numbers (K = 2–10). Model performance is evaluated using both perplexity and topic coherence. Perplexity scores are computed based on held-in likelihood, while coherence scores are calculated using the original tokenized texts and dictionary representation. All topic solutions within the specified range are retained, and the top 30 keywords for each topic under each topic number are extracted and reported, allowing for systematic comparison of thematic structures across alternative model specifications.

Instead of treating topics as predefined frames, the resulting topic distributions are interpreted as the issue dimension of news framing, reflecting how cross-Strait relations are structured and emphasized in global media discourse over time.

*(c) Sentiment Analysis of the Headlines.* To capture the evaluative dimension of news framing, this study analyzes the sentiment orientation of news headlines. Headlines are chosen because they function as highly condensed, interpretive cues that frequently encode evaluative judgments and emotional tone.

Instead of relying on lexicon-based sentiment tools such as VADER, which may be limited in handling political and geopolitical contexts, this study employs a comparative large language model (LLM)-based sentiment classification approach. Two independent LLMs, GPT-4.1-mini (OpenAI) and Grok-4 (xAI), were accessed via their respective web interfaces to classify each headline as positive, negative, or neutral with respect to the core event subject. To ensure reproducibility and account for potential model updates, all sentiment classification queries were conducted on December 9, 2025.

To further discern the sentiment orientation toward different news subjects, the core event subject, distinct from the grammatical subject, is manually identified and annotated with its associated country or region. For example, in the headline “Experts Say Arms Sale to Taiwan Answer Defense Needs, But Spur New Questions”, although “Experts” serves as the grammatical subject, the core event subject is “Arms Sale to Taiwan”, which is thus tagged with the Taiwan region.

Given the possibility of model-specific bias, a multi-stage validation procedure is implemented. First, sentiment classifications produced by the two LLMs are compared. For headlines where the two models yield inconsistent sentiment labels, the cases are subjected to manual review. Two researchers independently examine these headlines and jointly determine the final sentiment classification through discussion and consensus, thereby resolving ambiguity in evaluative interpretation.

To assess and enhance the reliability of LLM-based sentiment classification, a multi-stage validation procedure is implemented.

First, sentiment labels produced by the two LLMs are compared. Headlines are divided into two subsets: cases where the two LLMs produce identical sentiment labels (LLM consensus cases), and cases where the two models yield different labels (LLM disagreement cases).

For instances where divergent classifications emerged between ChatGPT and Grok, human annotation was introduced as an external gold standard to evaluate the reliability of model-based judgments. Within this framework, a sample was operationalized as a valid identification in the context of model-assisted sentiment recognition if either model’s output aligned with the human benchmark. This dual-model validation strategy was employed to mitigate systematic bias inherent in relying on a single algorithmic architecture. Empirical results indicate that for samples with inter-model disagreement, the proportion of cases where at least one model corroborated the human annotation reached 94.14%. Furthermore, by synthesizing a unified predictive label and comparing it against human coding, we obtained a Cohen’s kappa of 0.9021, demonstrating that model-assisted sentiment classification maintains robust alignment with human judgment even in scenarios characterized by high uncertainty.

Regarding the subsample where Grok and GPT reached a consensus, the study eschewed the assumption of unconditional reliability, instead implementing a stratified random sampling protocol for manual verification. Specifically, 150 news headlines were selected from the consensus pool which is balanced across sentiment categories, for rigorous human auditing to ascertain the empirical accuracy of the model agreement. The verification revealed an inter-rater agreement rate of 0.96 and a Cohen’s kappa of 0.94, suggesting that the observed model consensus is grounded in empirical consistency rather than stochastic alignment.

Building upon these validation outcomes, the final sentiment variable was constructed through a multi-stage integration process. For all manually annotated or audited samples, human judgment was prioritized as the definitive result. For the remaining data, model outputs were adopted as the final sentiment labels only if consensus was achieved between ChatGPT and Grok. A subsequent data audit confirmed the absence of any residual cases involving model disagreement without human intervention, thereby ensuring that unverified coding results were excluded from the analytical phase. Finally, by integrating the certainty of human-coded samples with the empirical accuracy of the consensus-based samples, a weighted accuracy estimation for the aggregate sentiment variable was calculated at 0.973. Through this systematic triangulation, the study achieves rigorous validation and robustness control of automated sentiment analysis while circumventing dependency on any single computational tool.

## 4 Research findings

### 4.1 The representation of global media in cross-Strait relations

#### 4.1.1 Evolution of global attention and coverage balance.

**Fig 1:**
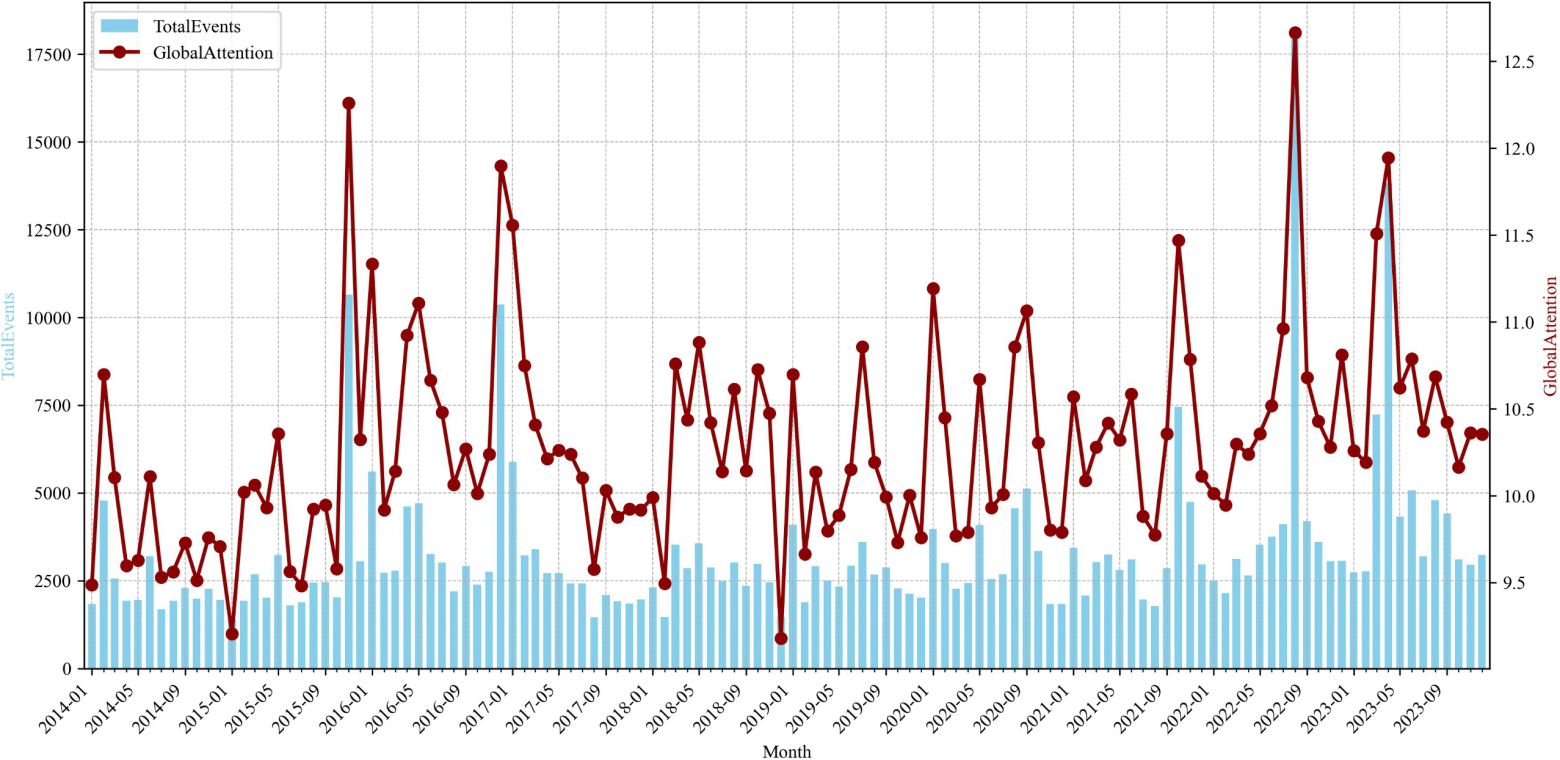
Monthly events and attention index of global reporting on cross-Strait relations.

The number of monthly reported events and attention index, which serves as an indicator of changes in global media attention (Fig 1). On an annual basis, the years 2016, 2022, and 2023 witnessed higher reporting volumes, each exceeding 40,000 events, with 2022 and 2023 surpassing 50,000 events, while other years recorded roughly 30,000 events. This pattern suggests that factors such as U.S.-China strategic competition and the Russo-Ukrainian war are reflected in the elevation of the Taiwan Strait as a hotspot of international focus, with the intensity of media attention still rising. At the monthly level, notable peaks occurred in November 2015, December 2016, August 2022, and April 2023. These peaks correspond respectively to significant events including the first Xi-Ma Meeting, the telephone call between President Trump and Tsai Ing-wen, Speaker Pelosi’s controversial visit to Taiwan, and Ma Ying-jeou’s visiting mainland China for ancestral worship, which demonstrates the global media’s sensitivity and timeliness in covering major cross-Strait events.

**Fig 2:**
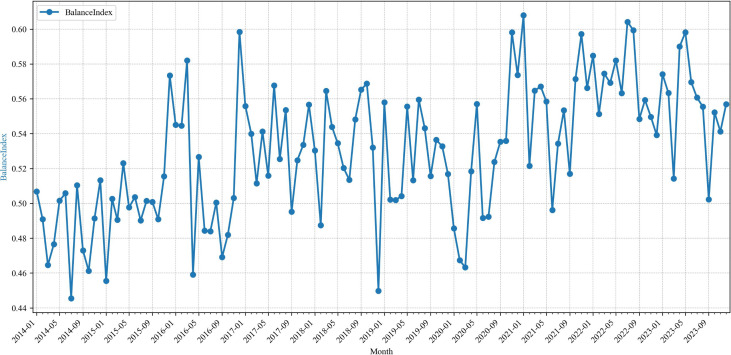
Balance index of global reporting on cross-Strait relations.

Over the sixty-month span from 2014 to 2023, the balance index exceeded 0.5 in 94 of the 120 months under review (Fig 2), indicating that, for the majority of this period, global media devoted greater attention to events initiated by the mainland China vis-à-vis Taiwan region rather than vice versa. Within the cross-Strait relations framing employed by international outlets, the mainland China consistently occupies the more more prominent role and is depicted as taking greater initiative in cross-Strait developments within global news narratives. This predominance is underpinned by mainland China’s expansive national capacity and its resolute pursuit of national reunification. The white paper “The Taiwan Question and China’s Reunification in the New Era” released in August 2022 declares that “resolving the Taiwan question and realizing China’s complete reunification is a shared aspiration of all the sons and daughters of the Chinese nation. It is indispensable for the realization of China’s rejuvenation” [48]. The mainland China has enshrined the “One China” principle as the non-negotiable precondition for establishing diplomatic relations. Amid intensifying great-power competition, mainland China has repeatedly cautioned the United States that the Taiwan issue represents a red line in Sino-U.S. relations, thereby underscoring its firm resolve.

Nevertheless, this does not imply that Taiwan merely adopts a reactive posture or that it is deprived of global media attention. Indeed, the balance index has ranged between a maximum of 0.608 in January 2021 and a minimum of 0.446 in July 2014, suggesting international reportage of cross-Strait affairs has remained relatively balanced, without manifest clear bias.

#### 4.1.2 Evolution of the impact index and tone index.

**Fig 3:**
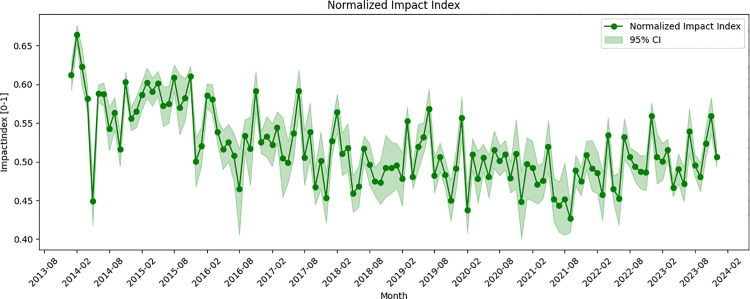
Evolution of the impact index of global media.

The Impact Index, derived from the GDELT’s GoldsteinScale, exhibits marked fluctuations in response to major cross-Strait events (Fig 3). In the aftermath of the 2014 Sunflower Movement, the index fell sharply before recovering during the Xi‑Ma meeting, and then declined again after the Democratic Progressive Party’s (DPP) election victory in early 2016.

Based on bootstrap resampling, February 2014 exhibits the highest estimated monthly Impact Index (mean = 0.664, 95% CI: [0.652–0.677]), indicating an unusually strong concentration of high-impact cross-Strait reporting during this period. This peak coincides with a series of high-level political interactions, coinciding with State Council Taiwan Affairs Office Director Zhang Zhijun’s inaugural ministerial meeting with Taiwan Mainland Affairs Council (MAC) Chair Wang Yu‑chi in Nanjing [49]. One week later, Xi Jinping received former Kuomintang (KMT) chairman Lien Chan at the Great Hall of the People in Beijing, where he advocated for peaceful development as part of China’s national rejuvenation agenda [50]. Conversely, lowest estimated monthly Impact Index (mean = 0.427, 95% CI: [0.409–0.445]) was recorded in September 2021, when Mainland Chinese planes flew into Taiwan Region’s air defense identification zone (ADIZ) after Han Kuang military exercises [51]. Moreover, Taiwan applied to join the Comprehensive and Progressive Agreement for Trans-Pacific Partnership (CPTPP) during that month, which elicited a public rebuke from Beijing [52]. These concurrent military and political pressures correspond with the pronounced trough in the Impact Index and highlight the complex challenges facing cross-Strait relations in 2021.

**Fig 4:**
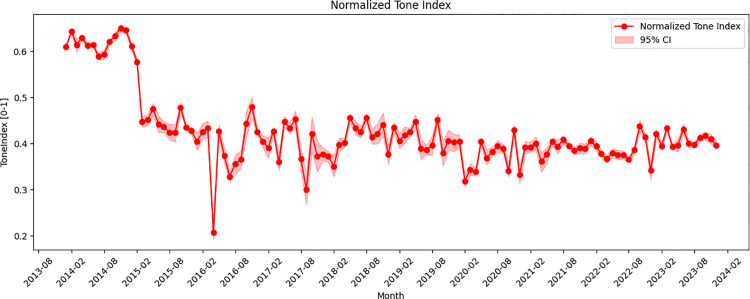
Evolution of the tone index of global media.

The global media’s Tone Index on cross-Strait relations displays notable fluctuation (**Fig 4**). Based on bootstrap estimates, beginning in March 2015, the overall sentiment of coverage progressively shifted toward a more negative orientation and remained at a relatively low level for an extended period, with the monthly Tone Index means consistently falling below the neutral midpoint of 0.5 thereafter. And November 2014 represents a local maximum in the Tone Index time series (mean = 0.650, 95% CI: [0.642–0.657]), whereas April 2016 corresponds to one of the lowest estimated values (mean = 0.207, 95% CI: [0.193–0.221]) over the 120-month period analyzed.

Both extremes are closely linked to significant political developments. The peak in November 2014 coincided with the 22nd APEC Economic Leaders’ Meeting held in Beijing, where Vincent Siew represented Taiwan under the designation “Chinese Taipei”. On November 9, Chinese President Xi Jinping met with Siew at the Great Hall of the People, reaffirmed the “1992 Consensus” and opposed “Taiwan independence”, emphasizing mutual trust and respect for divergent development paths as a foundation for long-term stability in cross-Strait relations [53]. This high-level engagement attracted substantial international media attention, contributing to the elevated Tone Index recorded that month.

In contrast, the trough in April 2016 corresponded to China’s extradition of 77 telecom fraud suspects from Kenya, including 45 Taiwanese for prosecution in the mainland [54]. This incident took place shortly before the inauguration of Tsai Ing-wen and was highly politicized by pro-independence media outlets in Taiwan. It was associated with a media storm and was widely interpreted by international outlets as a signal of rising cross-Strait tensions, contributing to the sharp decline in the Tone Index.

#### 4.1.3 Evolution of event categories.

**Fig 5:**
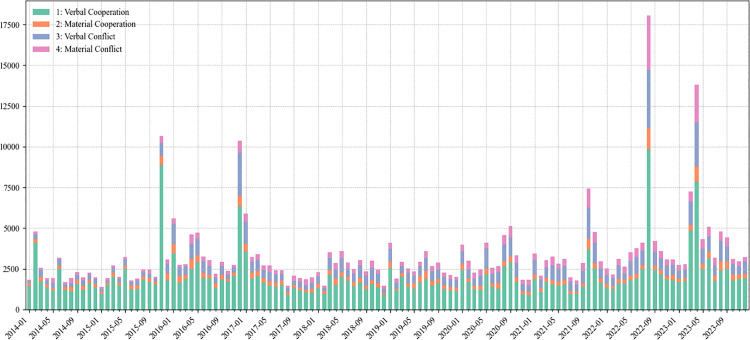
Evolution of the QuadClass on cross-Strait relations.

The evolution of the QuadClass of global media on cross-Strait shows that from the perspective of monthly fluctuations in cooperative and conflict events, November 2015, December 2016, August 2022, and April 2023 emerge as peak periods for cross-Strait cooperation, whereas December 2016, August 2022, and April 2023 similarly register heightened episodes of conflict (**Fig 5**). These convergence points in cooperation and confrontation generally coincide with significant political developments affecting both sides of the Taiwan Strait.

It is notable that cooperation and conflict events frequently co-occur within the same month. This seemingly paradoxical pattern likely results from the classification mechanism of GDELT’s CAMEO-based coding algorithm. Due to the limitations of natural language processing, the system relies principally on keyword matching to infer event typologies, leading at times to the incorrect classification of ostensibly cooperative actions reported in a negative context. For example, the December 2016 phone call between President Trump and Tsai Ing-wen was widely regarded as a negative incident, provoking strong protests from Beijing. Yet in the GDELT, media coverage of this event appears as a series of cooperative behaviors. The most frequent recorded event codes include “Consult”, “Make an appeal or request”, “Engage in negotiation”, “Make a visit”, “Make a statement”, and “Host a visit”. GDELT may interpret Beijing’s political appeals and public statements as evidence of cross-Strait cooperation, thereby producing a misleading cooperation narrative in the GDELT.

**Fig 6:**
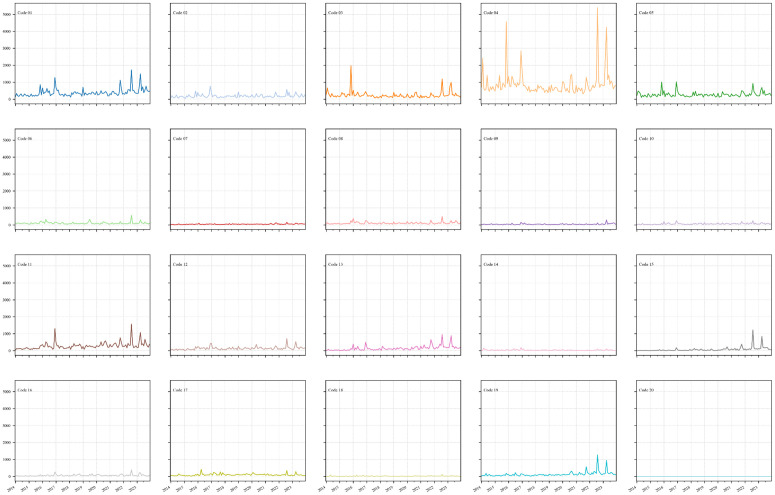
Evolution of the EventRootCode on cross-Strait relations.

The evolution of 20 different event categories on cross-Strait relations reported by global media illustrates that, during the decade, consultative and declaratory events have consistently dominated cross-Strait interactions (**Fig 6**). “Consult” (EventRootCode 04) ranks first in frequency, with pronounced peaks in November 2015, August 2022, and April 2023, reflecting moments when institutional dialogue was most vigorous. “Make public statement” (EventRootCode 01) and “Appeals” (EventRootCode 02) rank second and sixth in frequency. Their surges in December 2016, February 2018 (the trough) and August 2022 closely match the peaks in consultative events, suggesting that official pronouncements and public entreaties often precede or coincide with high‑level talks. “Engage in diplomatic cooperation” (EventRootCode 05) and “Express intent to cooperate” (EventRootCode 03) rank fourth and fifth. Their peak months almost perfectly overlap those of consultations, suggesting that once dialogue begins, both sides promptly move to formalize collaborative frameworks.

Meanwhile, “Engage in material cooperation” (EventRootCode 06) and “Provide aid” (EventRootCode 07) remain relatively infrequent yet exhibit a notable uptick in August 2022, implying that even amid heightened tension humanitarian and technical exchanges endure as limited safety-net channels. Other mid-frequency modalities, including “Yield” (EventRootCode 08) and “Demand” (EventRootCode 10), tend to track alongside material aid activities, serving as routine instruments within broader negotiation sequences.

In contrast, the conflict and deterrence cluster consists of “Disapprove” (EventRootCode 11), “Reject” (EventRootCode 12), “Threaten” (EventRootCode 13) and “Exhibit military posture” (EventRootCode 15). These events surged together in August 2022 and again in April 2023, coinciding with rises in “Reduce relations” (EventRootCode 16) and “Fight” (EventRootCode 19). This pattern illustrates how reports of provocations by pro-independence actors in Taiwan or external interventions are frequently accompanied by media-coded both diplomatic rebukes and demonstrations of force.

Nevertheless, extreme forms of violence such as “Engage in unconventional mass violence” (EventRootCode 20), “Assault” (EventRootCode 18) and “Protest” (EventRootCode 14) remain exceptionally rare. The global media representation indicates that despite occasional confrontations, the two sides have generally maintained a restrained, peace-oriented posture grounded in the “1992 Consensus” and a preference for resolving disputes through dialogue rather than coercion.

Overall, the media-recorded dynamics over the decade can be described as alternating phases of consultation, declaration and cooperation punctuated by periods of opposition and deterrence, yet consistently framed within a broader narrative of peaceful engagement.

### 4.2 Structural concentration and visibility patterns of high-impact media in cross-Strait reporting

#### 4.2.1 Structural visibility and event production among high-impact media.

Fifty high-impact media outlets were identified based on sustained event production in cross-Strait reporting (Table 1). These media collectively contributed 166,796 reports, accounting for 42.18% of all cross-Strait events in the dataset. Rather than representing a simple aggregation of prolific news producers, this subset constitutes a structurally salient stratum within the global news ecosystem, occupying a disproportionate share of informational visibility.

**Table 1:** High-impact media in cross-Strait reporting.

Domain	Number	Media	Nation/Region
taipeitimes.com	22325	Taipei times	Taiwan region
focustaiwan.tw	18775	Focus on Taiwan	Taiwan region
msn.com	13631	The Microsoft Network	United States
taiwannews.com.tw	7489	Taiwan news	Taiwan region
news.yahoo.com/yahoo.com	5656	Yahoo	United States
chinapost.com.tw	5120	The China Post	Taiwan region
theepochtimes.com	5094	Epoch Times	Taiwan region
eyeontaiwan.com	4848	Eye on Taiwan	Taiwan region
scmp.com	4586	South China Morning Post	Hong Kong
wantchinatimes.com	4567	Want China Times	Taiwan region
news.ltn.com.tw	4368	Liberty Times	Taiwan region
globalsecurity.org	4086	Global Security	United States
thediplomat.com	3185	The Diplomat	United States
dailymail.co.uk	3144	Daily Mail	The United Kingdom
straitstimes.com	2797	The Straits Times	Singapore
taiwansun.com	2750	Taiwan Sun	Taiwan region
nationalinterest.org	2434	The National Interest	United States
voanews.com	2279	Voice of America	United States
asia.nikkei.com	2277	Nikkei Asia	Japan
reuters.com	2236	Reuters	The United Kingdom
international.thenewslens.com	2116	The News Lens	Taiwan region
newsweek.com	2099	Newsweek	United States
globaltimes.cn	2042	Global Times	Mainland China
ecns.cn	1863	China News	Mainland China
bignewsnetwork.com	1812	Big News Network	The United Arab Emirates
rfa.org	1770	Radio Free Asia	United States
dw.com	1764	Deutsche Welle	Germany
japantimes.co.jp	1760	The Japan Times	Japan
express.co.uk	1695	Daily Expres	The United Kingdom
washingtonpost.com	1683	The Washington Post	United States
chinadaily.com.cn	1650	China Daily	Mainland China
nytimes.com	1593	New York Times	United States
china.org.cn	1576	China Internet Information Center	Mainland China
thestar.com.my	1565	The Star	Malaysia
breitbart.com	1546	Breitbart News	United States
menafn.com	1542	Menafn	Jordan
ibtimes.com	1512	International Business Times	United States
thestandard.com.hk	1479	The Standard	Hong Kong
sputniknews.com	1365	Sputnik news agency	Russia
foxnews.com	1299	Fox News	United States
theguardian.com	1293	The Guardian	The United Kingdom
en.people.cn	1244	People’s Daily	Mainland China
business-standard.com	1222	Business Standard	India
channelnewsasia.com	1147	Channel News Asia	Singapore
timesofindia.indiatimes.com	1136	The Times of India	India
aljazeera.com	1132	AL Jazeera	Qatar
news.xinhuanet.com	1117	Xinhua Net	Mainland China
economist.com	1073	The Economist	The United Kingdom
presstv.ir	1029	Press TV	Iran
article.wn.com	1025	World News Network	United States

A pronounced pattern of visibility concentration characterizes the global reporting structure. Although more than 8,000 unique media domains appear in the GDELT records of cross-Strait events, fewer than 1% of outlets account for over 40% of total coverage. This skewed distribution indicates that global narratives on cross-Strait relations are disproportionately represented by a relatively small number of media actors with sustained capacity for event reporting and international dissemination. Under such conditions, the internal dynamics among these high-impact outlets become analytically consequential.

The structural salience of high-impact media does not map neatly onto national power or institutional prominence. Mainland Chinese outlets, including China News Service, Global Times, China Daily, People’s Daily, and Xinhua News Agency, constitute only a limited share of total cross-Strait coverage, despite their organizational scale and state affiliation. This pattern reflects broader asymmetries in global communication, in which international visibility is unevenly distributed and shaped by transnational media infrastructures rather than solely by domestic media capacity.

Beyond the dominant Anglo-American and Taiwanese outlets, regional clusters of high-impact media emerge in Southeast Asia and the Middle East. Media organizations based in Singapore and Malaysia display sustained engagement with cross-Strait developments, reflecting regional security concerns and economic interdependence. Similarly, outlets from the United Arab Emirates, Jordan, and Qatar occupy visible positions within the dataset, suggesting efforts by non-Western actors to establish globally resonant news platforms within an otherwise uneven communication order.

Digital-native platforms and news aggregators further amplify the concentration of visibility. Outlets such as MSN, Yahoo News, Global Security Network, Breitbart News, and World News account for a substantial share of reporting volume, underscoring the growing role of platform-mediated dissemination in the circulation of global news flows. Importantly, the prominence of these outlets reinforces the centralization of attention around a limited number of nodes, rather than expanding discursive diversity.

These patterns indicate that global reporting on cross-Strait relations is characterized by a highly concentrated visibility structure. As a result, the question is no longer whether high-impact outlets matter, but how they differentiate their reporting practices within this structurally unequal environment. This structural condition motivates the subsequent analysis of internal differentiation among high-impact media, operationalized through the Deviation Index.

#### 4.2.2 Mapping structural differentiation among high-impact media via the deviation index.

After computing Deviation Index for high-impact media outlets, substantial heterogeneity emerges within this structurally salient group (Table 2). These differences unfold within a subset of outlets that already command disproportionate visibility in global reporting on cross-Strait relations, underscoring that concentrated attention does not entail narrative or structural homogeneity.

**Table 2:** The deviation index of high-impact media.

Dimension	Least Deviated Media (Top 5)	Index	Most Deviated Media (Top 5)	Index
**Event Count**	Want China Times	0.009	Press TV	0.925
	Eye on Taiwan	0.180	AL Jazeera	0.786
	The China Post	0.195	Menafn	0.786
	Xinhua Net	0.224	Taiwan Sun	0.779
	Channel News Asia	0.263	Big News Network	0.769
**Balance**	Want China Times	0.053	Fox News	0.806
	Taipei Times	0.086	The Times of India	0.780
	The China Post	0.092	Daily Express	0.763
	Focus on Taiwan	0.112	The Economist	0.757
	Eye on Taiwan	0.231	World News Network	0.754
**Impact**	Want China Times	0.077	The Economist	0.880
	Taipei Times	0.089	Daily Express	0.791
	Eye on Taiwan	0.107	The Washington Post	0.783
	The China Post	0.110	World News Network	0.781
	Focus on Taiwan	0.120	The Times of India	0.760
**Tone**	Want China Times	0.093	China Daily	0.923
	Taipei Times	0.160	People’s Daily	0.891
	Focus on Taiwan	0.228	China Internet Information Center	0.874
	The Microsoft Network	0.234	World News Network	0.848
	The National Interest	0.224	Xinhua Net	0.848
**QuadClass**	Taipei Times	0.122	The Economist	0.437
	Focus on Taiwan	0.153	The Times of India	0.416
	Eye on Taiwan	0.225	People’s Daily	0.411
	Taiwan news	0.226	Fox News	0.410
	The China Post	0.231	Business Standard	0.406
**EventRootCode**	Taipei Times	0.270	Business Standard	0.681
	Focus on Taiwan	0.305	The Economist	0.668
	The China Post	0.366	The Washington Post	0.662
	Want China Times	0.384	Radio Free Asia	0.662
	Eye on Taiwan	0.398	The Times of India	0.654
**Deviation Index**	Want China Times	0.148	World News Network	0.685
	Taipei times	0.178	The Economist	0.664
	Focus on Taiwan	0.212	People’s Daily	0.653
	The China Post	0.235	Fox News	0.646
	Eye on Taiwan	0.301	The Washington Post	0.611

The outlets with the lowest Deviation Index values, including Want China Times, Taipei Times, Focus on Taiwan, The China Post, and Eye on Taiwan, exhibit reporting profiles that closely approximate the aggregate global baseline across all six dimensions. This proximity does not imply neutrality or lack of editorial agency. Rather, it reflects a high degree of structural embeddedness within the prevailing equilibrium of global news production. As frequent contributors with sustained event volumes, these outlets participate directly in defining the statistical baseline, thereby reproducing dominant patterns of event distribution, tonal orientation, and issue framing within the global news ecosystem.

In contrast, the highest deviation index values occur for World News Network, The Economist, People’s Daily, Fox News and The Washington Post, indicating pronounced editorial divergence from the aggregate baseline. These deviations are not simply the result of limited coverage or occasional attention; rather, they indicate consistent differences in how highly visible media outlets select, frame, and emphasize cross-Strait events.

People’s Daily provides a particularly illustrative case of such internal differentiation. Over the ten‑year period, it recorded cross-Strait coverage in only 97 months, with fewer than ten events in 55 of those months, yielding a low EventCount score. Its mean balance index of 0.590 exceeds the global average of 0.539, reflecting a systematic emphasis on mainland‑initiated actions. The outlet’s QuadClass distribution further illustrates this orientation. Compared with the global baseline, People’s Daily allocates a substantially higher share of coverage to verbal cooperation (71.4% vs. 59.6%), while systematically underrepresenting material cooperation (5.9% vs. 9.0%), verbal conflict (16.6% vs. 19.9%), and material conflict (6.2% vs. 11.3%). On the continuous indices, its Impact Index is markedly higher than the global mean (1.149 vs. 0.352), whereas its Tone Index remains less negative (–1.223 vs. –1.767), indicating a relatively restrained affective framing. At the level of event typology, People’s Daily disproportionately emphasizes low-intensity, symbolic, and policy-oriented interactions. EventRootCode analysis shows that “Consult” (34.0%), “Make Public Statement” (14.2%), and “Express Intent to Cooperate” (7.0%) occur more frequently than in global coverage (25.5%, 11.2%, and 6.9%, respectively), while overtly confrontational actions, such as “Threaten” (2.4%), “Exhibit Military Posture” (0.5%), and “Fight”(1.6%), are consistently marginalized, each accounting for roughly half or less of their global proportions. These deviations reflect not episodic editorial selectivity but a stable and institutionally grounded reporting logic. As the official newspaper of the Chinese Communist Party, People’s Daily functions as a central policy communication instrument. Its coverage of cross-Strait relations prioritizes the signaling of Mainland China’s Taiwan policy, including emphasizing peace, stability, consultation, and national unity, while deliberately downplaying conflictual or militarized framings. This role helps explain why the outlet systematically exhibits a more positive and cooperative profile relative to the global reporting equilibrium: its primary function is not to mirror the diversity of international news narratives, but to articulate and disseminate the Mainland’s authoritative stance on cross-Strait relations within the global information environment.

Crucially, the Deviation Index captures differentiation independently of media influence as conventionally measured. Univariate and multivariate regression analyses reveal no statistically significant association between average NumMentions per event and any individual deviation dimension or the composite index. This finding confirms that editorial differentiation among high-impact media is not a function of prominence or visibility per se. Instead, it reflects how structurally salient actors exhibit selective patterns of attention, framing, and tone within a highly unequal communication environment.

The Deviation Index is statistically independent of media influence as measured by the average NumMentions per event. Uni-variate and multivariate regression analyses demonstrate no significant association between influence and any of the six deviation dimensions or their composite, indicating that editorial distinctiveness does not inherently correlate with an outlet’s prominence in cross-Strait reporting. This finding confirms that editorial differentiation among high-impact media is not a function of prominence or visibility per se. Instead, it reflects how structurally salient actors selectively organize attention, framing, and tone within a highly unequal communication environment.

In this sense, the Deviation Index does not rank outlets by influence or ideological extremity, but identifies the spectrum of editorial divergence among those already positioned at the center of global visibility. The coexistence of low-deviation stabilizers and high-deviation differentiators within the high-impact media cluster highlights the dual structure of global communication imbalance: while visibility is externally concentrated, narrative and editorial practices remain internally differentiated.

### 4.3 Framing cross-Strait relations in significant global news based on machine learning

#### 4.3.1 Issue-based framing of cross-Strait relations via thematic modeling.

By balancing perplexity and coherence, the optimal number of topics for each year was determined through manual review of the original texts and the summarization of thematic keywords (Table 3). The detailed keywords of the themes are presented in S1 Table of supporting information.

**Table 3:** Optimal number of topics, perplexity, coherence and themees for annual coverage.

Year	Optimal Number (Perplexity/Coherence)	Themes
**2014**	5 (181.136/ 0.406)	T1: Cross-strait issues amid South China Sea and regional disputes T2: Aviation disasters and large-scale commercial incidents involving Taiwan T3: Sino-US solar trade war and anti-dumping duties T4: Cross-Strait political relations and Sunflower student movement T5: Hong Kong democracy protests and social movements
**2015**	4 (155.756/ 0.495)	T1: The TransAsia Airways flight 235 crash in Taipei T2: The historic Ma-Xi meeting and regional diplomacy T3: U.S.-Taiwan defense ties and South China Sea disputes T4: 2016 presidential election build-up and party politics
**2016**	3 (175.837/ 0.375)	T1: Tsai Ing-wen’s inauguration and the Trump-Tsai phone call T2: South China Sea tensions, the Hsiung Feng III missile mishap and Sao Tome and Principe broke diplomatic ties with Taiwan T3: Cross-border telecom fraud and the Kenya deportation crisis
**2017**	6 (179.947/ 0.415)	T1: Great power games and Asia-Pacific security strategy T2: Human rights cases and civil society activism (the Lee Ming-che case) T3: Diplomatic isolation and cross-strait diplomatic tug-of-war T4: The Trump inauguration and domestic political transition T5: Digital governance and information integrity in Taiwan’s political communication T6: Foxconn’s investment in Wisconsin and economic diplomacy
**2018**	3 (178.171/ 0.395)	T1: 2018 local elections and the “Han Kuo-yu wave” T2: Cross-Strait military standoff and diplomatic sovereignty T3: Taiwan region faces strategic squeeze amid intensifying U.S.-China rivalry
**2019**	5 (208.432/ 0.339)	T1: Social governance, religious policy, and civil rights T2: Cross-strait political confrontation and competing claims of legitimacy T3: Sino-US trade friction and human rights diplomacy T4: Indo-Pacific strategic security and maritime presence T5: Hong Kong anti-extradition movement and its spillover effect
**2020**	4 (217.355/ 0.436)	T1: Global COVID-19 pandemic and cross-strait response T2: U.S.-Taiwan high-level visits and strategic alignment T3: Geopolitical security and regional economic rivalry T4: The Hong Kong national security law and its spillover to Taiwan
**2021**	4 (182.945/ 0.370)	T1: Global climate agenda and geopolitical shifts in the Pacific T2: Vaccine diplomacy and the cross-strait COVID-19 struggle T3: Mainland China’s ideological governance and regime legitimacy narratives T4: Escalating military tensions and Biden’s Taiwan policy
**2022**	4 (183.435/ 0.441)	T1: Global COVID-19 pandemic and cross-Strait response T2: U.S.-Taiwan high-level visits and strategic alignment T3: Geopolitical security and regional economic rivalry T4: The Hong Kong national security law and its spillover to Taiwan
**2023**	5 (179.932/ 0.382)	T1: Diplomatic tug-of-war and Ma Ying-jeou’s mainland visit T2: Tsai Ing-wen’s US transit and military countermeasures T3: Post-pandemic recovery and socio-economic challenges T4: Tech cold war, influence operations, and global sanctions T5: Semiconductor hegemony and strategic alliances

From 2014 to 2023, global media coverage of cross-Strait relations distilled into five dominant frames, each illustrated by representative topics: (1) event-centered conflict and risk framing, emphasizing crises like South China Sea disputes (2014T1), aviation disasters (2014T2; 2015T1), strategic squeezes amid U.S.-China rivalry (2018T3), and Pelosi’s visit framed as the catalyst for the Fourth Taiwan Strait Crisis (2022T4), illustrating the media’s tendency to prioritize high-impact events over routine interactions; (2) electoral politics and internal contestation framing, interpreting dynamics through Taiwan’s domestic lens via the Sunflower Student Movement (2014T4), election cycles (2015T4; 2018T1; 2019T2), and Tsai Ing-wen’s inauguration (2016T1), underscoring sovereignty debates’ evolution through democratic processes; (3) great-power rivalry and strategic instrumentalization framing, positioning Taiwan as a U.S.-China pivot in the Trump-Tsai call (2016T1), Indo-Pacific alignments (2018T3; 2019T4), high-level visits (2020T2), and military tensions (2021T4), de-emphasizing bilateral history for geopolitical signaling; (4) ideological governance and regime contrast framing, juxtaposing mainland control with Taiwan’s norms in human rights cases (2017T2; 2021T3) and Hong Kong events like protests (2014T5; 2019T5) and the National Security Law (2020T4), with declining prominence post-2020; and (5) functional interaction, disruption, and conditional reopening framing, addressing exchanges such as solar trade disputes (2014T3), Foxconn investments (2017T6), pandemic responses (2020T1; 2021T2), and post-pandemic diplomacy (2023T1; 2023T3), coexisting with security narratives. Collectively, these frames highlight a securitization shift influenced by Western biases, interplaying domestic, regional, and global factors in cross-Strait discourse.

First, major event reporting functions as a primary organizing logic through which cross-Strait relations are rendered visible to global audiences. Early coverage situates Taiwan-related issues within broader regional contexts, such as maritime disputes and regional security tensions, as in 2014T1, or frames Taiwan as a site of transnational risk through aviation disasters and commercial incidents, exemplified by 2014T2 and 2015T1. Nevertheless, Western media’s disproportionate share of coverage has led to a prevalence of conflict incidents in headlines, marginalizing themes such as Mainland China’s reunification policies, popular affinity across the Taiwan Strait, and international adherence to the “One China” principle.

Second, thematic emphasis shifts from non-security-centered issues in the earlier years to predominantly security- and sovereignty-focused narratives after the late 2010s. Before 2018, global reporting frequently approached cross-Strait relations through economic, industrial, and social lenses that did not place Taiwan at the center of military confrontation. Topics such as trade disputes in the solar industry in 2014T3 and Foxconn’s investment in the United States in 2017T6 portray Taiwan primarily as an economic actor embedded in global production and policy networks rather than as a focal point of strategic risk. From 2018 onward, however, military security, diplomatic confrontation, and great-power rivalry increasingly dominate global headlines. Taiwan is progressively framed as facing mounting external pressure amid intensified U.S.-China strategic competition, as illustrated by 2018T3 and subsequent coverage of high-level visits and security alignment in 2020T2. In this later period, economic and social themes do not disappear but are increasingly subordinated to security-oriented narratives. A limited reappearance of interaction-focused reporting occurs only in 2023, following post-pandemic reopening and symbolic cross-Strait engagements such as Ma Ying-jeou’s mainland visit in 2023T1.

Third, global media recurrently frame cross-Strait relations through Taiwan’s internal political contestation and its entanglement with broader ideological narratives. The 2014 Sunflower Movement, highlighted in 2014T4, marks a critical turning point by foregrounding societal apprehension toward economic integration with the mainland. Subsequent reporting increasingly depicts cross-Strait relations as filtered through electoral politics, partisan competition, and debates over democratic legitimacy, as seen in 2018T1 and 2019T2. Pro-independence forces are often portrayed as invoking Hong Kong as a rhetorical reference point, generating narratives that conflate Taiwan’s sovereignty debate with other regional autonomy movements, particularly in 2014T5 and 2019T5. Since 2020, however, the implementation of Hong Kong’s National Security Law and the decline of large-scale protest movements have reduced the prominence of this framing.

These frames demonstrate that global media coverage of cross-Strait relations from 2014 to 2023 has evolved from event-driven and low-politics reporting toward a predominantly high-politics narrative structured by electoral contestation, ideological contrast, and great-power rivalry. While moments of functional interaction and social exchange periodically re-enter the media agenda, they remain discursively secondary to conflict-oriented and strategic framings, producing a complex yet asymmetrically securitized portrayal of cross-Strait relations.

#### 4.3.2 Actor-sentiment framing in cross-Strait news headlines via LLM tools.

The principal actors and predominant sentiment categories appearing in the most consequential cross-Strait news headlines during the 2014–2023 period are also summarized (Table 4). Rather than merely reflecting episodic developments, these headline-level patterns reveal how global media selectively reconstruct the discursive representation of political reality through recurrent framing devices under conditions of intensified geopolitical mediatization. Three salient patterns emerge from this global media sample.

**Table 4:** Sentiment classification of headlines by nation/region.

Year	Nation/Region	Positive	Negative	Neutral	Total
**2014**	Mainland China and Hong Kong	15	14	9	38
	Taiwan Region	9	18	11	38
	USA	3	4	0	7
	Other	6	6	5	17
**2015**	Mainland China and Hong Kong	19	11	12	42
	Taiwan Region	21	20	23	64
	USA	0	1	2	3
	Other	2	0	6	8
**2016**	Mainland China and Hong Kong	3	21	11	34
	Taiwan Region	6	21	16	43
	USA	1	4	10	15
	Other	2	4	4	10
**2017**	Mainland China and Hong Kong	2	27	4	33
	Taiwan Region	6	17	12	36
	USA	5	7	13	25
	Other	1	2	4	7
**2018**	Mainland China and Hong Kong	3	22	5	30
	Taiwan Region	8	19	16	43
	USA	3	8	6	17
	Other	1	7	3	11
**2019**	Mainland China and Hong Kong	3	30	11	44
	Taiwan Region	4	10	19	33
	USA	4	6	10	19
	Other	1	0	5	6
**2020**	Mainland China and Hong Kong	2	29	7	38
	Taiwan Region	9	9	18	36
	USA	10	6	7	23
	Other	1	4	2	7
**2021**	Mainland China and Hong Kong	3	27	5	35
	Taiwan Region	6	14	3	23
	USA	13	12	10	35
	Other	3	3	6	12
**2022**	Mainland China and Hong Kong	5	25	9	39
	Taiwan Region	0	5	3	8
	USA	4	19	23	46
	Other	1	2	6	9
**2023**	Mainland China and Hong Kong	6	25	3	34
	Taiwan Region	5	14	7	26
	USA	9	8	11	28
	Other	6	3	6	15

Negative sentiment dominates the most consequential news headlines related to cross-Strait relations, whether the focus is Mainland China, Taiwan Region, or the United States, a large majority of affective terms are negative. From a framing perspective, this dominance should not be interpreted solely as a passive mirror of worsening relations, but as the interplay between real-world tensions and a media logic that privileges conflict, crisis, and threat as cognitively salient news frames. This prevalence reflects two convergent factors. First, the objective escalation of cross-Strait tensions, including Taipei’s policy reversals, intensified great-power involvement, and a higher frequency of confrontational incidents, has provided fertile discursive material for conflict-centered framing. Second, the inclusion of disaster reporting, most prominently the two TransAsia Airways crashes in July 2014 and February 2015, which together claimed over one hundred lives, including twenty‑eight mainland passengers, introduces non-political tragedy into the dataset; however, these events are nevertheless integrated into broader narratives of risk and instability surrounding cross-Strait interaction.

Actor focus in headline narratives has shifted markedly over the decade. In 2014–2016, references to Mainland China (inclusive of Hong Kong) and Taiwan appeared with comparable frequency, while US‑centric headlines were relatively rare. From 2017 onward, however, U.S.-focused headlines increased steadily and by 2021 surpassed those centered on Taiwan. This transformation signals a reconfiguration of dominant frames, in which cross-Strait relations are progressively subsumed under the broader interpretive schema of China-U.S. strategic rivalry.Rather than being framed primarily as a bilateral or intra-Chinese issue, the Taiwan Strait increasingly functions as a symbolic and strategic node within global power competition, reflecting the growing influence of international media logics that prioritize great-power confrontation over localized political dynamics.

The composition of other international actors has also evolved. Although constituting a smaller share of coverage, countries such as Japan, Canada, Australia, and India remain consistently present, indicating sustained interest in Taiwan‑related affairs. Their recurring visibility suggests that global media framing of the Taiwan issue extends beyond immediate stakeholders, embedding it within a wider network of alliance politics and normative positioning. Periodic attention to Taiwan’s diplomatic partners corresponds with episodes of diplomatic realignment, illustrating how moments of diplomatic rupture are selectively elevated as newsworthy signals of cross-Strait instability. After 2016, Southeast Asian states recede from headline prominence, likely reflecting a stabilization of Sino-ASEAN relations, particularly in trade, investment, and South China Sea cooperation, as well as a declining compatibility between routine regional cooperation and the conflict-oriented news frames favored by international media.

These patterns underscore how changes in geopolitical structure and media logic jointly condition sentiment distribution and actor visibility, structuring global news framing of the Taiwan issue in a manner consistent with the dynamics of mediated politics which refracts and interprets diplomatic reality.

## 5 Discussion

Based on nearly 400,000 cross-Strait events from the GDELT spanning 2014–2023, this study investigates three core research questions: the degree to which global media coverage reflects the evolution of cross-Strait relations; the regional balance and diversity of such reporting; and the longitudinal evolution of thematic frames and emotional tones within globally salient discourse. By combining large-scale event data with topic modeling and sentiment analysis, this study found that international news functions as both a mirror and a constitutive force, mediating the perception of political shifts through distinct framing patterns. The findings demonstrate that global reporting generally tracks real-world dynamics, such as leadership transitions or military crises, but also embeds these events within ideological and geopolitical narratives. By operationalizing “media logic” through quantifiable metrics, this study moves the mediatization framework from a descriptive concept to a diagnostic tool for international relations.

When compared with existing scholarship, these results confirm and refine prior understandings of media imbalance. Consistent with Guo and Vargo’s findings [7], this study corroborates the concentration of discourse power in Western and Taiwanese outlets, while mainland Chinese perspectives remain underrepresented. However, the data reveal a more dynamic process than previously documented: narrative tone and thematic emphasis are not fixed but fluctuate in response to Sino-U.S. strategic competition and regional electoral cycles. In contrast, prior studies tended to treat these representations as static ideological positions. The divergence between this study and earlier framing analyses may stem from three factors. First, the longitudinal design captures temporal variations that short-term case studies often overlook. Second, computational methods enable the detection of subtle tonal shifts across large volumes of data, revealing incremental redefinitions of the “Taiwan question” in global discourse. Third, this study’s cross-national scope exposes how regional political contexts condition media narratives, suggesting that cross-Strait framing is less a matter of bias than of structural dependency within global communication flows.

Interpreted through the theoretical lens of mediatized politics, these findings illustrate how cross-Strait relations have become increasingly influenced by media logic. The study confirms that news coverage does not simply reflect political events; it reconstructs them through selection, emphasis, and affective framing. This supports Mazzoleni and Schulz’s proposition that modern politics is “media-conditioned” rather than merely “media-covered” [3]. Moreover, in line with Brommesson and Ekengren [5], the results suggest that the mediatization of foreign policy extends to regional conflicts: the visibility, tone, and interpretive boundaries of cross-Strait issues are filtered through journalistic routines and global attention cycles. From this perspective, the asymmetry observed in global reporting is not only a question of discourse inequality but a structural outcome of mediatized international politics. By incorporating computational framing analysis into this theoretical paradigm, the present research contributes to bridging a critical gap: it operationalizes “media logic” as an empirical variable measurable through large-scale data.

Beyond its theoretical contribution, this study carries several practical implications. First, mainland Chinese media should strengthen their English-language communication capacity to project more balanced narratives about cross-Strait relations and reduce the dominance of Western and Taiwanese discourses in the international arena. Second, Western media should exercise caution in framing the Taiwan issue predominantly through conflict and crisis, as such coverage can reinforce perceptions of instability and potentially exacerbate the discursive climate of confrontation. Third, Taiwanese media should pursue more comprehensive and less partisan reporting, ensuring that journalistic practices do not amplify internal polarization at the expense of accuracy and fairness. Collectively, these suggestions emphasize that journalistic responsibility, transparency, and narrative diversity are essential to preventing the mediatized amplification of geopolitical tensions. Practically, these findings serve as a cautionary note for stakeholders: the observed “securitization” of cross-Strait discourse via global media logic reduces the perceived room for diplomatic maneuver, suggesting that managing the “mediatized” dimension of the conflict is an essential complement to managing its material military-security dimension.

At the same time, the research is still insufficient in some aspects, which needs future studies to explore. First, data completeness in GDELT is not absolute. In the analysis of high-impact media, it revealed that no event records for Want China Times, a publication affiliated with the China Times Media Group, appear after October 2015. Such omissions may skew aggregate measures of cross-Strait reporting balance and volume. Second, during sample selection, a few URLs provided by GDELT were defunct, preventing us from retrieving full-text articles for certain high-profile events. Researchers may consider integrating additional databases or regional archives to compensate for missing media sources. Third, the linguistic and structural bias inherent in GDELT, which reflects the existing global information hierarchy, means that our findings should be interpreted as the dominant global narrative rather than a demographically representative survey of all local perspectives. Finally, AI-generated sentiment analysis results for news headlines differ from lexicon-based sentiment models. For example, artificial intelligence occasionally interprets news headlines about the U.S. selling weapons to Taiwan as positive information, while large language models classify such events as negative. Future research could explore more precise and nuanced prompts, or employ other hybrid methods, to make AI’s analysis results closer to human judgment, thereby improving interpretability and accuracy.

## 6 Conclusion

This study systematically examined how global media outlets frame and interpret the evolution of cross-Strait relations over a ten-year period at both macro and micro levels. At the macro level, four continuous indices (Attention, Balance, Impact, and Tone), together with two distributional measures (QuadClass and EventRootCode) were constructed to depict how international outlets have framed shifts in cross‑Strait dynamics. These indices reveal that while the overall trajectory of cross‑Strait relations exhibits a downward trend, it is punctuated by notable fluctuations. In addition, a URL-based analysis identified the most influential media organizations in reporting cross-Strait events, uncovering a highly uneven distribution of discursive power and quantifying each outlet’s editorial orientation relative to the global baseline. At the micro level, a corpus of the 1000 significant headlines was subjected to topic modeling and sentiment classification to assess thematic emphases and affective valence. The results demonstrate a clear evolution from “low-politics” themes, such as trade and cultural exchange, toward “high-politics” issues, including security tensions and major power competition, while negative sentiment has consistently dominated global news headlines.

Theoretically, this study contributes to the mediatization of politics literature by demonstrating how cross-Strait relations function as a mediated political reality which is increasingly intertwined with media logic rather than merely reflected by it. First, by analyzing 395,465 GDELT‑coded news events and multi‑dimensional indices (Attention, Balance, Impact, Tone, QuadClass, EventRootCode), it demonstrates that large-scale news big‑data can dynamically map the evolution of cross‑Strait relations. The peaks and troughs of these indicators record the major events that occurred on both sides of the Taiwan Strait over the past decade and their subsequent impacts. Second, the study reveals a clear asymmetry in the visibility and interpretive framing of the Taiwan issue. About 40% of cross-Strait news events are reported by a very small number of media outlets, most of which are concentrated in East Asia, Europe and the United States. Taiwanese and Western media dominate the narrative of the Taiwan Strait issue, while mainland Chinese media remain relatively marginalized, illustrating the structural imbalance of global communication power that mediates how geopolitical legitimacy is negotiated in the public sphere. Third, the study demonstrates that editorial uniqueness, rather than audience reach, is a significant factor in narrative divergence, as evidenced by the People’s Daily, which emphasizes cooperative and positive frames to construct a stable image of cross-Strait ties.

At the disciplinary level, the study provides an empirical and methodological blueprint for integrating big-data analytics with computational text mining in global communication research. First, the combined use of longitudinal macro indicators and micro-level topic modeling illustrates a promising path for bridging large-scale structural mapping and interpretive depth. This methodological design operationalizes “media logic” in measurable terms, allowing the mediatization framework to move from theoretical abstraction to empirical testing. Second, the GDELT dataset’s temporal coverage and global scope enable predictive applications for regional stability. For example, forecasting peace or conflict trends through event frequencies and tonal patterns, as in prior studies that estimated global “peace indices” using media data. Future research could extend this predictive modeling to measure the influence of third-party actors, such as the United States or Japan, on the evolution of cross-Strait relations, thereby clarifying how external powers are represented within and influence mediated geopolitical narratives.

In conclusion, this study underscores that cross-Strait relations are not only a geopolitical reality but a mediatized construct negotiated through by global communication structures. The scientific significance of this research lies in its robust operationalization of mediatization theory, providing a scalable computational framework to measure how international news systems co-construct the perception of political realities. Practically, by identifying the systemic shift from “low politics” to “high politics,” the findings offer critical insights for policymakers into how regional tensions are securitized and amplified within the global information ecosystem. Within the wider disciplinary context of political communication and international relations, this study bridges the gap between structural media power and geopolitical perception, demonstrating that in an age of global interdependence, the struggle for discursive legitimacy increasingly frames the competition for material power. By integrating the mediatization of politics framework with computational social science methods, this study provides both theoretical innovation and empirical evidence for how international news systems actively mediate the representation of political events and reconfigure the discursive landscape of contemporary diplomacy.

## Supporting information

S1 Table The detailed keywords of the themes (2014–2023). Top 30 keywords of each themes via LDA model (in Section 4.3.1) are presented in this table.
(DOC)

## References

[1] Stares PB. Preventive priorities survey 2025. New York. 2025. https://cdn.cfr.org/sites/default/files/report_pdf/Preventive%20Priorities%20Survey%202025.pdf

[2] Qiang X, Wang J. Parallel perceptions: divergent perspectives of the United States and China on the Taiwan issue and risky implications. China Rev. 2023;23:41–76.

[3] Mazzoleni G, Schulz W. “Mediatization” of Politics: A Challenge for Democracy?. Political Communication. 1999;16(3):247–61. doi: 10.1080/105846099198613

[4] Couldry N, Hepp A. Conceptualizing Mediatization: Contexts, Traditions, Arguments. Commun Theor. 2013;23(3):191–202. doi: 10.1111/comt.12019

[5] Brommesson D, Ekengren A-M. The Mediatization of Foreign Policy, Political Decision-Making, and Humanitarian Intervention. Palgrave Macmillan US. 2017. doi: 10.1057/978-1-137-54461-2

[6] Pan H-H, Wu W-C, Chang Y-T. How Chinese Citizens Perceive Cross-Strait Relations: Survey Results from Ten Major Cities in China. Journal of Contemporary China. 2017;26(106):616–31. doi: 10.1080/10670564.2017.1274835

[7] Guo L, Vargo CJ. Global Intermedia Agenda Setting: A Big Data Analysis of International News Flow. J Commun. 2017;67(4):499–520. doi: 10.1111/jcom.12311

[8] Wu CKS. How public opinion shapes taiwan’s sunflower movement. J East Asian Stud. 2019;19(3):289–307. doi: 10.1017/jea.2019.30

[9] Wu J. 10. Taiwan’s Sunflower Occupy Movement as a Transformative Resistance to the “China Factor”. In: Lee CK, Sing M. Take Back Our Future. Cornell University Press. 2019. 215–40. doi: 10.7591/9781501740930-011

[10] Effendi TD. Mapping the cross-strait studies as a specific research field. China Contemp Polit Stud. 2020;26(2–3):113–31.

[11] Jaw-Nian H. The China Factor in Taiwan’s Media. chinaperspectives. 2017;2017(3):27–36. doi: 10.4000/chinaperspectives.7388

[12] Pan XL, Qiao TZ. The selection and construction of the news: A comparative study of the news reports between Taiwan and the mainland on Lian Chan’s journey of the peace. Journal of Journalism & Communication Studies. 2005;(04):54–65.

[13] Yang HJ. A discourse analysis of news reports on a trip to Taiwan by mainland Chinese real estate moguls by the United Daily News, China Times and Liberty Times. Journal of Leader University. 2010;7(1):81–99. doi: 10.7080/JLU.201011.0081

[14] Huang YL, Qiu YX. Fractured imagination of Taiwan’s national identity: A framing analysis of media coverage on “Xi-Ma meeting”. Journalism Research. 2016;135(1):129–35.

[15] Ruiz Casado JA, Lock N. Imaginaries of enmity across the Taiwan Strait: The ‘cartoon war’ between Taipei Times and Global Times. China Information. 2025;39(1):75–109. doi: 10.1177/0920203x241307626

[16] Lams L. Cross-Strait relations from a linguistic perspective. In: Damm J, Lim P. European perspectives on Taiwan. Wiesbaden: VS Verlag für Sozialwissenschaften. 2012. 196–214.

[17] Hsu SP. Cross-Strait relations in the wake of Taiwan’s 2022 local elections. Am J Chin Stud. 2023;:81–94.

[18] Liu A, Li X. Assessing Public Support for (Non-)Peaceful Unification with Taiwan: Evidence from a Nationwide Survey in China. SSRN Journal. 2023. doi: 10.2139/ssrn.4381723

[19] Chen C-JJ, Zheng V. Changing Attitudes toward China in Taiwan and Hong Kong in the Xi Jinping Era. Journal of Contemporary China. 2021;31(134):250–66. doi: 10.1080/10670564.2021.1945738

[20] Lu X. Discourse and ideology: The Taiwan issue in the Chinese and American media. Research and practice in professional discourse. 2022;:589–608.

[21] Chen ZY. On the Preference of Taiwan-related News by Japanese Mainstream Media from the Perspective of Intertextuality. Journal of Nanjing Xiaozhuang University. 2020;36(2):98–103.

[22] Nylén P. Japanese newspapers and representations of Taiwan: A discourse analysis of the depiction of Taiwan in the newspaper editorials of Asahi Shimbun and Yomiuri Shimbun between 1990-2017. Stockholm: Stockholm University. 2017.

[23] Calello MC, Chen HR. Taiwan, China and Central American Allies: A Discourse Analysis of the Costa Rican Diplomatic Shift News Coverage. Taiwan International Studies Quarterly. 2013;9(1):139–77.

[24] Yoo T. Analysis of Uzbekistan’s relations with China, Russia, and South Korea: utilizing text mining based on gdelt big data. corr. 2024;02(04):1–7. doi: 10.55640/corr-v02i04-01

[25] Sun X. Research on the impact of international public opinion on China’s outward foreign direct investment—a case study of countries along the Belt and Road. In: International Journal of Frontiers in Sociology. 2024;6(6):43–9. doi: 10.25236/IJFS.2024.060608

[26] Schiller HI. Communication and Cultural Domination. Routledge. 2019. doi: 10.4324/9781315179162

[27] Shamsuddin M. The New World Information Order. Pakistan Horizon, 1987; 40(1), 80–94.

[28] Golan GJ, Himelboim I. Can World System Theory predict news flow on twitter? The case of government-sponsored broadcasting. Information, Communication & Society. 2015;19(8):1150–70. doi: 10.1080/1369118x.2015.1106572

[29] Gunaratne SA. Prospects and Limitations of World System Theory for Media Analysis. Gazette (Leiden, Netherlands). 2001;63(2–3):121–48. doi: 10.1177/0016549201063002003

[30] Bhatti SJ, Billinson PP, Cornell LA, Das A, Gammon C, Kelly LO, et al. A country comparative analysis of international print media’s framing of the COVID-19 pandemic. Int J Commun. 2022;16(27).

[31] Sullivan J, Lee DS. Soft Power Runs into Popular Geopolitics: Western Media Frames Democratic Taiwan. Int J Taiwan Stud. 2018;1(2):273–300. doi: 10.1163/24688800-00102003

[32] Hsu C-J. China’s Influence on Taiwan’s Media. Asian Survey. 2014;54(3):515–39. doi: 10.1525/as.2014.54.3.515

[33] Stern S, Livan G, Smith RE. A network perspective on intermedia agenda-setting. Appl Netw Sci. 2020;5(1). doi: 10.1007/s41109-020-00272-4

[34] Horne BD, Nørregaard J, Adali S. Different spirals of sameness: A study of content sharing in mainstream and alternative media. 2019. https://arxiv.org/abs/1904.01534

[35] Pan JS, Qi WH, Wang ZC, Lyu HJ, Luo JB. Bias or diversity? Unraveling fine-grained thematic discrepancy in U.S. news headlines. 2023. https://workshop-proceedings.icwsm.org/pdf/2023_25.pdf

[36] Thussu D. Changing Geopolitics of Global Communication. Routledge. 2024. doi: 10.4324/9781315271699

[37] Goffman E. Frame analysis: An essay on the organization of experience. Cambridge (MA): Harvard University Press. 1974.

[38] Entman RM. Framing: Toward Clarification of a Fractured Paradigm. Journal of Communication. 1993;43(4):51–8. doi: 10.1111/j.1460-2466.1993.tb01304.x

[39] Roberts ME, Stewart BM, Tingley D, Lucas C, Leder-Luis J, Gadarian SK. Structural Topic Models for Open-Ended Survey Responses. American Journal of Political Science. 2014;58(4):1064–82.

[40] Tenenboim-Weinblatt K, Baden C. Journalistic transformation: How source texts are turned into news stories. Journalism. 2016;19(4):481–99. doi: 10.1177/1464884916667873

[41] Maier D, Waldherr A, Miltner P, Wiedemann G, Niekler A, Keinert A, et al. Applying LDA Topic Modeling in Communication Research: Toward a Valid and Reliable Methodology. Communication Methods and Measures. 2018;12(2–3):93–118. doi: 10.1080/19312458.2018.1430754

[42] Balahur A, Hermida JM, Montoyo A. Detecting implicit expressions of emotion in text: A comparative analysis. Decision Support Systems. 2012;53(4):742–53. doi: 10.1016/j.dss.2012.05.024

[43] Liu B. Sentiment analysis: mining opinions, sentiments, and emotions. Cambridge: Cambridge University Press. 2015.

[44] Burscher B, Vliegenthart R, de Vreese CH. Frames Beyond Words. Social Science Computer Review. 2016;34(5):530–45. doi: 10.1177/0894439315596385

[45] Ali M, Hassan N. A Survey of Computational Framing Analysis Approaches. In: Proceedings of the 2022 Conference on Empirical Methods in Natural Language Processing, 2022. 9335–48. doi: 10.18653/v1/2022.emnlp-main.633

[46] Matthes J. What’s in a Frame? A Content Analysis of Media Framing Studies in the World’s Leading Communication Journals, 1990-2005. Journalism & Mass Communication Quarterly. 2009;86(2):349–67. doi: 10.1177/107769900908600206

[47] Esser F, Strömbäck J. Mediatization of politics: understanding the transformation of western democracies. London: Palgrave Macmillan. 2014. doi: 10.1177/1464884915570535

[48] The State Council Information Office of the People’s Republic of China. The Taiwan question and China’s reunification in the new era.2022. http://english.scio.gov.cn/whitepapers/2022-08/10/content_78365819_2.htm

[49] Tiezzi S. China, Taiwan to hold first high-level meeting since 1949. The Diplomat. 2014. https://thediplomat.com/2014/01/china-taiwan-to-hold-first-high-level-meeting-since-1949/

[50] Xinhua. Xi calls for mutual trust, respect across Taiwan Strait. People’s Daily. 2014. https://en.people.cn/n/2014/1110/c102839-8807015.html

[51] Taipei Times. Chinese planes spotted as military exercises end. Taipei Times. 2021. https://www.taipeitimes.com/News/taiwan/archives/2021/09/18/2003764570

[52] Li L, Cheng TF. China blasts Taiwan’s bid to join CPTPP trade pact. Nikkei Asia. 2021. https://asia.nikkei.com/Economy/Trade/China-blasts-Taiwan-s-bid-to-join-CPTPP-trade-pact

[53] Xinhua. Xi calls for mutual trust, respect across Taiwan Strait. People’s Daily. 2014. https://en.people.cn/n/2014/1110/c102839-8807015.html

[54] Luu C. Chinese officials: 45 Taiwanese “confess” to alleged telecom fraud. CNN. 2016. https://edition.cnn.com/2016/04/22/asia/china-taiwan-deportation

