# Supplementary material for: Mapping the evolution of cross-Strait relations via global news big data (2014–2023): An analysis integrating GDELT and machine learning
Source: PLoS One. 2026 Feb 13;21(2):e0342755. doi: 10.1371/journal.pone.0342755 (PMC12904412; doi:10.1371/journal.pone.0342755)
Supplement: S1 Table — Top 30 keywords of each themes via LDA model (in Section 4.3.1) are presented in this table. (DOC) [file pone.0342755.s001.doc]

**Supporting Information**

**S1 Table. The Detailed Keywords of the Themes (2014-2023).**

| **Year** | **Top 30 Keywords of each Theme** |
| --- | --- |
| 2014 | Topic 1: Cross-Strait Issues amid South China Sea and Regional Disputes Theme Words: china, taiwan, chinese, island, vietnam, taiwanese, people, plane, south_china_sea, phillippine, beijing, flight, wednesday, penghu, military, oil, crashed, ministry, claim, rig, vietnamese, transasia, agency, weather, water, official, news, foreign, ship, land |
|  | Topic 2: Aviation Disasters and Large-scale Commercial Incidents Involving Taiwan Theme Words: crash, dead, air, china, boeing, people, airline, 747, airbus, plane, dupont, airport, killing, 12, billion, lng, liew, chinese, deal, landing, export, takeoff, a300, company, project, 4, gas, 20, economic, ocean |
|  | Topic 3: Sino-US Solar Trade War and Anti-dumping Duties Theme Words: solar, chinese, import, duty, industry, panel, china, trade, percent, united_states, product, anti, commerce, solarworld, drug, taiwan, battery, manufacturer, government, department, market, complaint, country, india, cell, ion, washington, ruling, power, domestic |
|  | Topic 4: Cross-Strait Political Relations and Sunflower Student Movement Theme Words: taiwan, china, beijing, chinese, island, trade, president, mainland, government, ma, official, hong_kong, party, relation, political, talk, taiwanese, people, meeting, taipei, agreement, zhang, tie, war, student, ma_ying, jeou, protest, economic, pact |
|  | Topic 5: Hong Kong Democracy Protests and Social Movements Theme Words: hong_kong, china, blackberry, people, beijing, business, mainland, company, protest, democracy, country, protester, police, chinese, government, chen, taiwan, phone, leader, city, device, day, party, communist, share, system, security, square, wednesday, billion |
| 2015 | Topic 1: The TransAsia Airways Flight 235 Crash in Taipei Theme Words: engine, pilot, plane, flight, crash, taiwan, river, people, taipei, atr, transasia, aircraft, 72, crew, crashed, takeoff, passenger, accident, shut, taiwanese, airway, left, investigation, control, keelung, wednesday, 4, rescue, airline, missing |
|  | Topic 2: The Historic Ma-Xi Meeting and Regional Diplomacy Theme Words: china, chinese, singapore, vietnam, war, people, government, republic, time, history, president, island, talk, taiwan, power, student, wu, lai, ministry, minister, relationship, country, japan, vietnamese, party, public, life, family, handshake, lin |
|  | Topic 3: U.S.-Taiwan Defense Ties and South China Sea Disputes Theme Words: taiwan, china, military, beijing, chinese, island, force, sale, united_states, relation, told, political, war, tie, president, washington, party, policy, foreign, ministry, news, ma, taiwanese, ruled, trade, issue, exercise, dispute, taipei, mainland |
|  | Topic 4: 2016 Presidential Election Build-up and Party Politics Theme Words: taiwan, china, ma, meeting, beijing, president, party, chinese, nationalist, relation, leader, island, xi, office, mainland, election, taiwanese, strait, taipei, cross, war, government, jeou, ma_ying, presidential, political, communist, bank, civil, opposition |
| 2016 | Topic 1: Tsai Ing-wen's Inauguration and the Trump-Tsai Phone Call Theme Words: taiwan, china, president, beijing, tsai, trump, call, chinese, policy, relation, island, taiwanese, leader, elect, office, mainland, tie, independence, government, diplomatic, party, election, war, wen, relationship, tsai_ing, phone, country, foreign, statement |
|  | Topic 2: South China Sea Tensions, the Hsiung Feng III Missile Mishap and São Tomé and Príncipe broke diplomatic ties with Taiwan Theme Words: china, taiwan, island, missile, official, beijing, claim, sao, tome, people, south_china_sea, water, government, country, navy, chinese, military, lawyer, boat, ministry, told, sea, taiwanese, fishing, nation, incident, international, ship, phillippine, indigenous |
|  | Topic 3: Cross-border Telecom Fraud and the Kenya Deportation Crisis Theme Words: china, taiwan, chinese, taiwanese, mainland, beijing, kenya, suspect, official, people, island, student, fraud, ministry, deported, international, council, government, robot, police, right, citizen, malasia, north, law, country, affair, news, deportation, authority |
| 2017 | Topic 1: Great Power Games and Asia-Pacific Security Strategy Theme Words: china, trump, president, chinese, beijing, taiwan, military, island, policy, north, xi, korea, south_china_sea, claim, call, leader, force, foreign, official, international, administration, country, election, security, time, trade, asia, nuclear, journal, united_states |
|  | Topic 2: Human Rights Cases and Civil Society Activism (The Lee Ming-che Case) Theme Words: china, lee, taiwan, chinese, mainland, activist, government, right, hong_kong, political, authority, beijing, law, ming, tibetan, che, independence, taiwanese, people, democracy, human, party, city, pro, president, power, march, security, trial, freedom |
|  | Topic 3: Diplomatic Isolation and Cross-Strait Diplomatic Tug-of-War Theme Words: taiwan, china, beijing, tsai, president, island, relation, taiwanese, trump, chinese, diplomatic, government, taipei, tie, country, official, foreign, international, ministry, leader, ally, meeting, office, policy, wen, month, visit, tsai_ing, mainland, united_states |
|  | Topic 4: The Trump Inauguration and Domestic Political Transition Theme Words: trump, president, donald_trump, inauguration, police, american, america, washington, people, clinton, india, parade, 12, campaign, inaugural, country, protester, 1, office, nation, time, white, presidential, administration, national, capitol, barack_obama, dog, leader, presidency |
|  | Topic 5: Digital governance and information integrity in Taiwan’s political communication Theme Words: tang, taiwan, president, people, ma, government, public, post, office, don, twitter, chen, time, country, chinese, medium, democracy, taiwanese, news, issue, internet, system, minister, rumor, idea, computer, digital, fake, social, leader |
|  | Topic 6: Foxconn’s Investment in Wisconsin and Economic Diplomacy Theme Words: trump, president, house, elect, white, donald_trump, foxconn, intelligence, united_states, job, director, china, wednesday, chinese, republican, wisconsin, factory, official, national, company, democratic, time, election, committee, clayton, meeting, 3, democrat, plant, political |
| 2018 | Topic 1: 2018 Local Elections and the “Han Kuo-yu Wave” Theme Words: taiwan, china, tsai, beijing, election, dpp, island, independence, taipei, taiwanese, vote, chinese, nationalist, diplomatic, government, saturday, voter, mainland, referendum, party, pro, people, country, local, film, support, international, official, relation, kaohsiung |
|  | Topic 2: Cross-Strait Military Standoff and Diplomatic Sovereignty Theme Words: taiwan, china, beijing, island, chinese, president, tsai, diplomatic, country, force, relation, government, military, tie, drill, foreign, ally, taiwanese, mainland, airline, wen, territory, taipei, ministry, tsai_ing, independence, war, people, Tsai_Ing-wen, month |
|  | Topic 3: Taiwan Region Faces Strategic Squeeze amid Intensifying U.S.–China Rivalry Theme Words: china, taiwan, chinese, beijing, island, military, president, country, official, government, tie, south_china_sea, relation, taiwanese, dominican_republic, international, diplomatic, missile, united_states, trade, force, trump, taipei, washington, tsai, people, policy, nation, time, mattis |
| 2019 | Topic 1: Social Governance, Religious Policy, and Civil Rights Theme Words: china, church, government, taiwan, service, chinese, party, liao, success, people, told, train, time, 100, ren, right, delay, lgbt, marriage, day, organization, artist, 10, religious, religion, family, detained, month, johnston, rain |
|  | Topic 2: Cross-Strait political confrontation and competing claims of legitimacy Theme Words: taiwan, china, beijing, chinese, island, hong_kong, president, tsai, military, government, country, force, mainland, people, taiwanese, party, official, democracy, protest, sale, election, united_states, wen, law, taiepi, political, war, support, arm, relation |
|  | Topic 3: Sino-US Trade Friction and Human Rights Diplomacy Theme Words: china, chinese, trade, people, beijing, tibet, soybean, government, event, tibetan, time, february, foreign, including, right, american, city, canadian, united_states, official, president, held, party, minister, country, human, student, union, political, anniversary |
|  | Topic 4: Indo-Pacific Strategic Security and Maritime Presence Theme Words: china, military, island, pacific, taiwan, war, region, solomon, beijing, chinese, nation, report, economic, washington, country, south_china_sea, climate, change, power, united_states, diplomatic, relation, security, trade, tie, government, strategic, president, carrier, prc |
|  | Topic 5: Hong Kong Anti-Extradition Movement and Its Spillover Effect Theme Words: hong_kong, taiwan, china, taiwanese, government, protest, extradition, leader, city, visit, law, amendment, chan, storm, suspect, prison, beijing, island, country, japan, haiti, typhoon, chinese, pro, mainland, project, police, including, saturday, lee |
| 2020 | Topic 1: Global COVID-19 Pandemic and Cross-Strait Response Theme Words: health, china, people, coronavirus, outbreak, virus, wuhan, reported, country, taiwan, pandemic, official, response, death, chinese, confirmed, city, spread, disease, covid, 19, day, tuesday, government, week, mask, public, time, authority, human |
|  | Topic 2: U.S.-Taiwan High-Level Visits and Strategic Alignment Theme Words: taiwan, china, beijing, island, chinese, tsai, official, taiwanese, president, hong_kong, government, visit, country, democracy, military, force, people, relation, taiepi, washington, health, election, support, international, foreign, sale, azar, ministry, security, united_states |
|  | Topic 3: Geopolitical Security and Regional Economic Rivalry Theme Words: china, chinese, beijing, country, military, report, 1, taiwan, xi, region, cee, nuclear, president, party, official, business, billion, missile, government, zhou, economic, security, political, trade, people, mask, eu, trump, central, coronavirus |
|  | Topic 4: The Hong Kong National Security Law and its Spillover to Taiwan Theme Words: china, hong_kong, pizza, xi, biden, chinese, beijing, national, taiwan, security, ship, country, system, law, people, government, news, meeting, global, kite, foreign, legislation, ramen, leader, south_china_sea, white, pro, hut, time, including |
| 2021 | Topic 1: Global Climate Agenda and Geopolitical Shifts in the Pacific Theme Words: xi, leader, 20, biden, summit, climate, putin, russia, pod, sogavare, country, people, usky, president, russian, person, french, european, concern, decision, nuclear, international, company, transport, solomon, honiara, agenda, speed, fishing, family |
|  | Topic 2: Vaccine Diplomacy and the Cross-Strait COVID-19 Struggle Theme Words: taiwan, vaccine, china, country, dos, chinese, 19, japan, island, covid, beijing, people, shot, government, biontech, party, health, pandemic, political, foreign, taipei, xi, australia, deal, day, force, international, pressure, global, taiwanese |
|  | Topic 3: Mainland China’s Ideological Governance and Regime Legitimacy Narratives Theme Words: party, china, xi, student, office, communist, hong_kong, college, pompeo, chinese, country, people, university, community, government, rule, leader, political, european, democracy, restriction, federal, policy, speech, power, employee, school, petition, control, billion |
|  | Topic 4: Escalating Military Tensions and Biden’s Taiwan Policy Theme Words: taiwan, china, island, military, chinese, beijing, biden, president, official, administration, force, defense, government, relation, people, foreign, united_states, day, support, claim, washington, pacific, air, including, statement, territory, taipei, issue, aircraft, war |
| 2022 | Topic 1: Pandemic Policy Pivot and Global Travel Dynamics Theme Words: china, covid, test, country, people, virus, 19, city, chinese, infection, traveler, travel, government, hong_kong, korea, south, global, variant, japan, blinken, announced, foreign, official, economy, testing, health, pandemic, meeting, day, week |
|  | Topic 2: Strategic Convergence of the Ukraine Crisis and Taiwan Strait Theme Words: china, russia, taiwan, president, ukraine, biden, policy, military, chinese, time, call, xi, beijing, cambodia, administration, police, war, house, support, greene, putin, document, al, white, issue, question, strategic, department, crisis, invasion |
|  | Topic 3: Energy Security, Climate Politics, and Social Unrest Theme Words: people, power, california, xie, energy, plant, asean, lawmaker, newsom, day, governor, protest, increase, chou, told, chinese, cnn, diablo, week, hong_kong, ago, record, anti, news, democracy, heat, grid, church, price, political |
|  | Topic 4: Pelosi's Visit and the Fourth Taiwan Strait Crisis Theme Words: taiwan, china, visit, pelosi, chinese, military, beijing, island, government, united_states, official, president, foreign, force, missile, taiwan_strait, country, support, relation, policy, exercise, region, house, drill, washington, people, war, taiwanese, ministry, trip |
| 2023 | Topic 1: Diplomatic Tug-of-War and Ma Ying-jeou's Mainland Visit Theme Words: china, taiwan, chinese, beijing, honduras, government, military, tie, taiwanese, diplomatic, country, ma, relation, president, taipei, philippine, defense, visit, force, america, ally, city, people, foreign, trip, island, latin, nation, zone, wu |
|  | Topic 2: Tsai Ing-wen's US Transit and Military Countermeasures Theme Words: taiwan, china, beijing, chinese, island, military, visit, official, president, tsai, meeting, united_states, force, relation, foreign, country, washington, defense, ministry, government, taiwan_strait, war, house, told, wednesday, national, security, people, independence, taiwanese |
|  | Topic 3: Post-Pandemic Recovery and Socio-Economic Challenges Theme Words: beijing, visit, mainland, day, city, hong_kong, shanghai, china, 7, ma, quarter, protest, temperature, rate, trillion, taiwan, kishida, march, 3, 2019, rule, strict, student, leader, debt, exchange, national, chinese, japanese, university |
|  | Topic 4: Tech Cold War, Influence Operations, and Global Sanctions Theme Words: china, russia, chinese, official, country, taiwan, operation, tesla, influence, minister, huawei, beijing, people, chip, graphika, blinken, park, company, ukraine, government, sanction, weapon, nuclear, american, development, global, g7, shooting, news, medium |
|  | Topic 5: Semiconductor Hegemony and Strategic Alliances Theme Words: china, taiwan, trade, beijing, chinese, russia, country, economic, military, xi, ukraine, chip, putin, marijuana, taiwanese, semiconductor, australia, island, leader, visit, support, effort, relation, security, albanese, war, billion, agreement, people, cable |
